# Longitudinal profiles of parental self- and child-focused emotion regulation

**DOI:** 10.1038/s44271-026-00469-w

**Published:** 2026-05-16

**Authors:** Gesine Jordan, Kristina Stockinger, Christine Schiltz, Samuel Greiff, Ziwen Teuber

**Affiliations:** 1 Department of Behavioral and Cognitive Sciences, University of Luxembourg, Esch-sur Alzette, Luxembourg; 2 Department of Psychology, University of Augsburg, Augsburg, Germany; 3 School of Social Sciences & Technology and Centre for International Student Assessment (ZIB), Technical University Munich, Munich, Germany

**Keywords:** Human behaviour, Risk factors, Development studies

## Abstract

Parenting is an emotion-eliciting experience that requires parents to navigate the dual demands of regulating their own and their children’s emotions, drawing on regulatory strategies such as reappraisal and rumination. However, patterns of self- and child-focused reappraisal and rumination and their associations with parental exhaustion, children’s internalizing problems, children’s academic achievement, and parent–child relationships remain underexplored. Our preregistered three-wave online study targeted U.S. parents of sixth–ninth graders (*N* = 1,046; 51.53% mothers; *M*_age_ = 43.22). We employed latent profile analyses and latent transition analyses. Results revealed three profiles of parental self- and child-focused emotion regulation strategies, stable across a 4-month timeframe: *Reappraisers* (T_1_ ≈ 9%, T_2_ ≈ 7%) showed high use of self- and child-focused reappraisal, *self-ruminators* (T_1_ ≈ 25%, T_2_ ≈ 31%) reported high self-focused rumination and low use of other strategies, and *average regulators* (T_1_ ≈ 66%, T_2_ ≈ 61%) showed moderate use of all strategies. In addition, *reappraisers* reported the lowest levels of parental exhaustion and children’s internalizing problems and the highest parent–child relationship closeness. By contrast, *self-ruminators* reported the highest levels of parental exhaustion and children’s internalizing problems and the lowest parent–child relationship closeness. However, profile membership did not predict children’s academic achievement. The study provides important insights into distinct subgroups of parents on the basis of their combined use of self- and child-focused emotion regulation strategies, highlighting the differential relations of these strategies on parental well-being and child outcomes.

## Abstract (web summary)

In a longitudinal study, three profiles of parental self- and child-focused reappraisal and rumination were identified that are associated with parental exhaustion, children’s internalizing problems, and parent–child relationship closeness.

## Longitudinal profiles of parental self- and child-focused emotion regulation

Parenting involves navigating the dual challenges of managing one’s own emotions while also helping children regulate theirs. To balance these demands, parents often rely on their habitual use of *emotion regulation* strategies, which may be primarily directed towards their own emotions (*self-focused emotion regulation*) or their child’s emotions (*child-focused emotion regulation*). Two influential strategies for shaping the emotion generation process are (a) *reappraisal*, the reinterpretation of a situation to alter its meaning, often in a more positive or goal-congruent direction^1–3^, as well as (b) *rumination*, characterized by persistent, repetitive, and distress-focused thoughts^1,4,5^, and a response style also related to psychopathology (e.g., depression and anxiety disorders)^4,6^. Parental self- and child-focused reappraisal and rumination are associated with parental well-being, children’s emotional development, and children’s adjustment^7,8^, with early to mid-adolescence representing a less clearly understood period regarding parental emotional support^9^. Understanding patterns of parental self- and child-focused emotion regulation has important empirical and practical implications because different combinations may help explain variability in parental and child well-being, as well as the parent–child relationship, thereby informing the development of tailored interventions. Thus, we used latent profile and latent transition analyses to investigate habitual patterns of self- and child-focused emotion regulation in parents of sixth–ninth graders as well as profile stability over a period of 4 months at the beginning of the school year. Furthermore, we examined correlates of these profiles including parents’ educational level, parents’ gender, parental exhaustion, children’s internalizing problems, children’s achievement, and parent–child relationship closeness.

Consider, for instance, an adolescent who comes home upset after receiving a poor report card and is facing the possibility of repeating the year. The parent may attempt to regulate their own negative emotions (e.g., disappointment or frustration) while also helping the child downregulate their emotional distress. These mechanisms are classified under the concept of emotion regulation, which refers to the process by which individuals modify emotional states in pursuit of specific goals^10,11^. Such regulatory goals can be primarily directed towards one’s own emotions (self-focused emotion regulation) or another person’s emotions (other-focused emotion regulation)^12^, with these goal directions being activated separately or coactivated. The literature on parental self- and child-focused emotion regulation is closely intertwined with overlapping concepts such as coregulation, emotion socialization and interpersonal emotion regulation^13^, where prior research indicates interrelations between how parents regulate their own and their children’s emotions^14,15^. More precisely, parents with higher self-focused emotion regulation skills or capacities are more likely to respond supportively to children’s emotions, tend to be more involved and sensitive in their parenting, and use fewer negative and unsupportive practices, including hostility and harshness^8,16–18^. Conversely, parents who engage more in self-focused emotion dysregulation tend to use more non-supportive and disengaged regulation practices and are more likely to engage in child maltreatment^8,17,19–22^.

Parents’ self- and child-focused emotion regulation facilitate children’s emotion regulation development and adjustment^9,23,24^, as reflected in the Tripartite Model through the mechanisms of *observation* and *parenting practices*^7^. Observation refers to the process by which children learn from observing and imitating their parents’ emotional behaviors (e.g., parents’ self-focused emotion regulation), whereas parenting practices describe parents’ direct responses to their children’s emotions, including child-focused emotion regulation. Children’s need for parental support varies across development, and as they enter adolescence, they increasingly seek greater autonomy, influence how parents regulate their emotions, and draw on self-regulation^25,26^. Nonetheless, parents remain an important source of emotional support, and their nurturance is associated with higher well-being in their adolescent child^9,27^. During early and mid-adolescence, parental regulatory efforts adapt to children’s changing needs, and parenting tasks shift towards fostering adolescents’ independence^26,27^. Consequently, direct support (e.g., child-focused emotion regulation) likely becomes more tailored to critical developmental tasks, such as helping adolescents manage academic and performance-related pressures^28^. At the same time, parents’ self-focused emotion regulation remains an important source of social learning through modeling^7,29^. Given the high prevalence of negative emotions in emotion regulation goals and the importance of achievement contexts in adolescence^28,30^, the present study focused on child-focused emotion regulation during children’s negative achievement emotions. Our sample comprised parents of children in Grades 6–9 because (a) early to mid-adolescence is marked by biological changes that make this stage a particularly emotionally vulnerable one requiring parental support^27,31,32^, and (b) after the transition to secondary education, academic demands and maladaptive patterns of emotional development increase, on average^33,34^.

Considering the earlier example of a child being upset about a poor report card, a parent might regulate their own emotions by reminding themselves that this situation does not define their child’s future (self-focused reappraisal) or by dwelling on thoughts such as “I must be doing something wrong as a parent” (self-focused rumination). In the child-focused context, the parent could help the child reinterpret the setback as an opportunity to learn (child-focused reappraisal) or direct the child’s attention toward the failure by repeatedly questioning what went wrong (child-focused rumination). Reappraisal and rumination regulate early stages in the emotion-generation process before response modulations, with reappraisal being categorized under processes of cognitive change and rumination under attentional deployment in the extended process model of emotion regulation^1,2,11^. Habitually, higher levels of self-focused reappraisal are often associated with higher well-being, stress recovery, and psychological adjustment^35^, whereas higher levels of self-focused rumination are associated with increased negative mood and psychopathology, including symptoms of depression and anxiety^4,6^.

In everyday parenting life, however, reappraisal and rumination can sometimes deviate from their habitual associations and may be helpful in some situations and unhelpful in others^12^. Despite the consistent benefits of self-focused reappraisal, it can, for example, be less effective in situations of high-emotionality or when cognitive resources are limited^36–38^. Self-focused rumination, on the other hand, has been described by individuals as a strategy to process and reflect on negative emotions to feel more in control of a situation^4,39^. Nevertheless, given its close link to disorders such as depression, the intense self-reflection involved in self-focused rumination can indicate individuals’ avoidance of situations or responsibilities, and often occurs unconsciously or involuntarily^4,6,39^. However, most research on reappraisal and rumination has been conducted in self-focused contexts, and the extent to which results can be applied to interpersonal contexts, especially parenting, is unclear. Longitudinal profiles of parental reappraisal and rumination can tackle questions on their coexistence, address how such coexistence in turn is related to family correlates, and reveal habitual differences in the use of self- and child-focused strategies.

Evidence on self- and child-focused emotion regulation has been primarily derived from variable-centered methods, even though heterogeneity in emotion regulation strategy use and parenting practices is evident^8,40^. Individuals differ in their use of self-focused emotion regulation strategies and parenting practices depending on factors such as context and personality^40–42^. Latent profile analysis and latent transition analysis are methodologies that allow researchers to identify how different emotion regulation strategies co-occur and form interrelated patterns within individuals over time, rather than focusing only on associations between single emotion regulation dimensions and parent and child outcomes^43^. Identifying profiles of parental reappraisal and rumination can capture the interconnectivity of self- and child-focused emotion regulation strategies, thereby clarifying concurrent use of these strategies in relation to antecedents and outcomes.

Variable-centered findings suggest that parents tend to use similar strategies when regulating their own and their children’s emotions^44^. Similarly, parents with higher self-focused emotion regulation skills are more likely to engage in warmer and more constructive parenting practices^8,27,40^. Therefore, some profiles might be characterized by high self- and child-focused reappraisal and low self- and child-focused rumination, or the reverse. Interestingly, self-focused emotion regulation is not consistently associated with parenting behaviors^8,40^, which may be reflected in subgroups of parents showing misalignment between self- and child-focused emotion regulation. For example, a parent who frequently engages in self-focused rumination and finds that it increases their own negative feelings may try to protect their child from similar experiences by avoiding child-focused rumination. Subgroups might also reveal the co-occurrence of parents’ reappraisal and rumination use^45,46^, where longitudinal patterns are lacking to date.

Historically, emotion regulation and parenting practices have been regarded as stable trait-like characteristics, with reappraisal and rumination reflecting learned responses to distress^3,47,48^. Self-focused reappraisal and rumination have shown moderate stability, although substantial individual variability has also been reported, with some individuals demonstrating stable patterns and others changing over time^1,49^. In recent years, flexible changes in emotion regulation and parenting practices have been highlighted^40,50^, and stability in parents’ self- and child-focused emotion regulation can be related to parental situational stressors and emotions as well as changes in children’s needs^51,52^. Understanding transitions in parental emotion regulation profiles can clarify how stable or malleable these patterns are, disentangle their roles in parent and child correlates, and identify at-risk groups (e.g., groups of parents or children with lower well-being).

Profiles of parent self- and child-focused emotion regulation may be associated with sociodemographic factors such as parental gender and educational level. Stereotypically, mothers’ parenting practices are more strongly associated with emotionality and nurturance in Western cultures^53^, although evidence of parents’ emotion regulation is mixed, with some studies indicating no differences in parents’ emotional socialization^54^, and others suggesting higher involvement of mothers including more supportive strategies^55–57^. Self-focused rumination is also consistently higher among females than males^4,48^, whereas in self- and child-focused reappraisal, gender differences are not reported as often^3,58–60^. Another sociodemographic variable that potentially predicts parental emotion regulation profiles is parents’ educational level, an indicator of socioeconomic status (SES), which is robustly associated with individuals’ health^61,62^. Parents likely also experience more stress and reduced well-being when experiencing financial hardship^40^, which may in turn be related to parents’ strategies for self- and child-focused emotion regulation.

Patterns of parents’ self- and child-focused emotion regulation are likely interconnected with family well-being and relationship closeness, with prior work predominantly focusing on links to child development^8,18,63^. Parents’ self-focused emotion regulation and parenting practices are robustly tied to how children regulate their own emotions, which in turn is closely linked to their internalizing problems, characterized by inwardly oriented affective disruptions^7,40,64,65^. Overall, parental self- and child-focused emotion regulation has been repeatedly associated with child adjustment^8^, which presumably encompasses academic achievement during adolescence^31,46,66^. Less attention has been given to parental correlates including parental exhaustion (i.e., persistent parenting-related stress and an early stage of parental burnout^67–69^), but evidence links parental exhaustion to lower self-focused reappraisal, higher self-focused rumination, and disruptions in child development^70–73^. Lastly, parent–child relationship closeness may be associated with parental self-and child-focused emotion regulation, as higher emotion regulation skills are related to more secure attachment^74,75^, and authoritative parenting characterized by warmth and structure^27,76^, whereas self-focused emotional instability has been linked to parental distancing and fewer or lower quality parent–child interactions^21,77^.

The aim of the current study was to identify subgroups of parents’ self- and child-focused emotion regulation (RQ1) and investigate their stability (RQ2). We expected profiles characterized by high self- and child-focused reappraisal combined with low self- and child-focused rumination and, conversely, low reappraisal combined with high rumination. Furthermore, we expected parents to show relatively stable patterns in their habitual use of self- and child-focused emotion regulation over time. In RQ3, we investigated associations between emotion regulation profiles and parent and child correlates. We hypothesized that the identified profiles would vary by parents’ educational level and gender. However, given the exploratory nature of our analyses and the mixed findings in the literature regarding group differences in reappraisal and rumination^3,48,59^, we did not specify the direction of these differences. Furthermore, we hypothesized that profile membership would predict parental exhaustion, children’s internalizing problems, children’s academic achievement, and the parent–child relationship. Profiles characterized by higher levels of self- and child-focused reappraisal were expected to be associated with lower parental exhaustion and children’s internalizing problems but higher children’s academic achievement and parent–child relationship closeness. By contrast, profiles characterized by greater use of self- and child-focused rumination were expected to be associated with higher parental exhaustion and children’s internalizing problems and lower children’s academic achievement and parent–child relationship closeness.

## Methods

### Participants and procedure

We collected longitudinal data at three time points (T_1_ in September 2023, T_2_ in January 2024, T_3_ in May 2024), following parents for one school year. After cleaning the data and excluding nonmatchable records, 1,373 participants completed the survey at T_1_, and 1,046 participants provided data at T_1_ and T_2_, indicating an attrition rate of 23.82% (*n* = 327). Thus, the final sample for the main analyses (T_1_–T_2_ dataset) consisted of *N* = 1046 parents of sixth–ninth graders residing in the US. At the third time point, 875 participants could be matched to the T_1_–T_2_ dataset. The percentage of missing values in the T_1_–T_2_ dataset was 0.60%, and in the dataset including all three time points the percentage was 4.62%. Participants’ mean age was *M*_*age*_ = 43.22 (*SD* = 9.35), and they reported that their child’s mean age was *M*_*age*_ = 11.78 (*SD* = 2.94). For feasibility reasons, we aimed to include one parent per family via Prolific and Lime survey (https://www.limesurvey.org). The sample was balanced between mothers and fathers with 539 participants (51.53%) identifying as female. Participants’ identified race was for 69.12% white (*n* = 723), 17.59% African American/Black (*n* = 184), 4.40% Asian (*n* = 46), 4.88% Mixed (*n* = 51), 2.29% Other (*n* = 24), and for 1.72% race was not reported (*n* = 18). Sixteen participants (1.53%) reported that their highest educational level was below secondary school, 215 (20.55%) reported secondary school, 130 (12.43%) reported an associate’s degree, 449 (42.93%) reported a bachelor’s degree, 184 (17.59%) reported a master’s degree, 25 (2.39%) reported a PhD, and 27 (2.58%) reported another level of education. Additionally, we coded households’ Highest International Socio-Economic Index of Occupational Status (HISEI)^78,79^, which had a mean of 64.65 (*SD* = 16.95, min = 15.35, max = 88.70). We recorded parents’ relationship status, with 871 parents responding to be married or in a committed relationship (83.27%), and 175 responding not to be (16.73%). Of parents married or in a committed relationship, 844 reported living in the same household as their partner (96.90%), 27 reported they did not (3.10%). Throughout the questionnaire, participants were instructed to refer to their youngest child in Grades 6–9. According to the parent report, 476 of these children identified as female (45.47%), 570 identified as male (54.42%), and none identified as gender diverse. In the sample, 1010 parents reported living in the same household as their child (96.56%), whereas 36 reported they did not (3.44%). Parents were prescreened to have at least one child in Grades 6 to 9. In the main study, 29.83% of the children were reported to be in Grade 6 (*n* = 312), 18.55% in Grade 7 (*n* = 194), 16.93% in Grade 8 (*n* = 177), 21.13% in Grade 9 (*n* = 221), and for 13.58% the grade was not indicated (*n* = 142). A total of *n* = 237 participants (22.66%) indicated that their child had special needs under *the Individuals with Disabilities Education Act*^80^ categories.

The study was approved by the Ethics Review Panel of the University of Luxembourg (Protocol number: ERP 23-061). Before participating, all parents provided informed consent in accordance with relevant guidelines. Participants were compensated via Prolific with $3 for the first wave, $4 for the second wave, and $6.46 for the third wave, with the latter including a bonus for participating in all three waves. The study design, research questions, and hypotheses were preregistered prior to data collection on August 30, 2023, on the Open Science Framework (OSF); see https://osf.io/4hvtj (RQ1, Hypothesis 2). At T_1_ and T_2_, a panel design was used, and the primary instruments relevant to the current study were administered identically in the two waves. At T_3_, the questionnaire was revised, and only two newly added outcomes from that wave (i.e., children’s internalizing problems and the parent–child relationship) were included in the present analyses, which were not preregistered. Because the preregistration included all research questions of the panel study *Predictors and Outcomes of Parental Burnout and Parental Educational Involvement*^81^, we report here only those relevant to the present paper. All data and codes are available on the OSF; see https://osf.io/ybt46/. In the following, we describe all the measures, the statistical analyses, how we determined our sample size, and all data exclusions.

### Measures

#### Demographic and socioeconomic variables

Parents’ and children’s demographic and socioeconomic variables were reported by parents in Prolific and Lime survey. Parents’ gender, as listed on official documents, was recorded by Prolific with 1 = *male*, 2 = *female*. Children’s gender was reported through Lime survey with 1 = *male*, 2 = *female*, and *3* = *other/diverse*. In addition, parents indicated their highest educational level ranging from 1 (*lower than secondary school*) to 6 (*PhD*).

#### Emotion regulation strategies

Two parental self-focused emotion regulation strategies were assessed, namely, self-focused reappraisal and self-focused rumination. Self-focused reappraisal was measured with the Emotion Regulation Questionnaire (ERQ^3^). We included four of the six items from the reappraisal subscale, specifically those aligned with the present study’s emotion goal of downregulating negative emotions, for example, “*I control my emotions by changing the way I think about the situation I’m in*” with a 5-point Likert response format ranging from 1 (*strongly disagree*) to 5 (*strongly agree*). The reliability of the subscale was very good with Cronbach’s α = 0.85 (T_1_) and 0.87 (T_2_). To assess self-focused rumination, the five-item brooding subscale from the Ruminative Responses Scale (RRS^5^) was used. An example item was “*Why do I have problems other people don’t have?”* Participants responded on a 5-point Likert scale ranging from 1 (*never*) to 5 (*always*). Cronbach’s α was good at both time points (α = 0.88 at T_1_ and 0.90 at T_2_). No specific timeframe was specified in the instructions to measure the habitual use of all emotion regulation strategies.

Additionally, we measured parents’ child-focused reappraisal and rumination to assess how parents attempt to regulate their children’s emotions, using the respective subscales from the Parental Assistance with Child Emotion Regulation (PACER) Questionnaire^82^. To investigate these processes in achievement-related contexts, the item stems of the scales were adapted to a situation in which the child’s negative emotions arose from receiving a poor grade on an important exam. All items were measured on a 5-point Likert scale ranging from 1 (*strongly disagree*) to 5 (*strongly agree*) and each subscale consisted of five items. The items of child-focused reappraisal included “*I help [adolescent’s nickname] change their feelings by thinking differently about their current situation*”, “*I help [adolescent’s nickname] think of a situation in a positive light*”, “*I help [adolescent’s nickname] see the situation from a different perspective*”, “*I help [adolescent’s nickname] try to see the positive aspects of a situation that is making them have negative feelings*”, and “*I encourage [adolescent’s nickname] to think of the positive side to their negative feelings*”. The items of child-focused rumination, on the other hand, included “*I help [adolescent’s nickname] replay the experience of negative feelings again and again in their mind*”, “*I help [adolescent’s nickname] replay whatever is making them have negative feelings*”, “*I help [adolescent’s nickname] think again and again about whatever is making them have negative feelings*”, “*I encourage [adolescent’s nickname] to think over and over again about why they are having negative feelings*”, and “*I help [adolescent’s nickname] think about situations that are upsetting or that cause negative feelings over and over again*”. The reliability was good for both subscales with α = 0.84 (T_1_) and .86 (T_2_) for child-focused reappraisal and α = 0.90 (T_1_) and 0.89 (T_2_) for child-focused rumination.

#### Parental exhaustion

Parental exhaustion was assessed using the exhaustion in parental role subscale of the Parental Burnout Assessment (PBA^73^), with a 6-point Likert scale ranging from 1 (*never*) to 6 (*every day*). The subscale was chosen on the basis of theoretical considerations, as parental exhaustion is considered an early warning sign and precursor of parental burnout^67^. Parental exhaustion was measured with nine items (e.g., “*I feel completely run down by my role as a parent*”). The subscale’s reliability was very good with α = 0.94 (T_1_) and 0.95 (T_2_), and the scale was positively skewed at both time points (skewness_T1_ = 1.44, skewness_T2_ = 1.30).

#### Children’s academic achievement

Parents reported their children’s academic achievement by indicating final grades from the last report card in Math, Science, and English. Participants chose the grading scales (e.g., GPA, letter grade), which were standardized into a letter-grade system ranging from 1 (*lowest academic achievement, equal to F*) to 13 (*highest academic achievement, equal to A* + ). The scale was negatively skewed at both time points (skewness_T1_ = −1.30, skewness_T2_ = −1.30). An individual mean score of all grades was included in the analyses to indicate the children’s overall academic achievement.

#### Children’s internalizing problems

Children’s internalizing problems were assessed using the 10-item internalizing problems score from the Strengths and Difficulties Questionnaire (SDQ^83^). The SDQ internalizing problems score is the sum of two subscales, Emotional Symptoms and Peer Relationship Problems, each consisting of five items. Selection of this score was theoretically driven, as internalizing problems are associated with children’s self-focused emotion regulation^63^ and therefore important to investigate in the context of parenting emotion regulation profiles. The score assessed the frequency of emotional symptoms (e.g., *restless, overactive, cannot stay still for long*) and the frequency of peer relationship problems (e.g., *often lies or cheats*). The response format ranged from 0 (*not true*) to 2 (*certainly true*). Cronbach’s α indicated good results with α = 0.80 (T_3_), and the scale was positively skewed with skewness_T3_ = 1.01.

#### Parent–child relationship

To measure the closeness of the parent–child relationship, the closeness subscale from the Child–Parent Relationship Scale – Short Form (CPRS-SF^84^) was selected. Four items from the subscale were chosen on the basis of their factor loadings (e.g., *I share an affectionate, warm relationship with [adolescent’s nickname]*). Responses were assessed on a 5-point Likert scale and ranged from 1 (*strongly disagree*) to 5 (*strongly agree*), and the internal consistency was good with α = 0.83 (T_3_). The scale was negatively skewed with skewness_T3_ = −1.24.

### Data analysis

Data analyses were computed with R^85^ (Version 4.4.2) and M*plus*^86^ (Version 8.6). We utilized the R packages *psych*^87^, *lavaan*^88^, *MplusAutomation*^89^*, haven*^90^, *car*^91^, and *ggplot2*^92^. The analyses primarily focused on data from T_1_ and T_2_, although two outcomes (parent–child relationship and children’s internalizing problems) absent from earlier assessments were included from T_3_. A priori power analyses were computed in R using Monte Carlo simulations. Results for the latent profile and transition analyses indicated that a total sample size of *N* = 500 was sufficient for all analyses, which was successfully attained. Oversampling was applied to reach the target sample size in a longitudinal panel design. In the following, data analyses are described on the basis of a prior study using a longitudinal person-oriented approach^93^ and a methodological guide^43^.

#### Longitudinal measurement invariance

First, cross-sectional Confirmatory Factor Analysis (CFA) models were tested for self- and child-focused emotion regulation strategies (four-factor CFA), parental exhaustion (one-factor CFA), children’s internalizing problems (two-factor CFA), and the parent–child relationship (one-factor CFA) to investigate construct validity. Afterwards, Longitudinal Measurement Invariance (LMI) models were estimated for parental self- and child-focused emotion regulation strategies using robust maximum likelihood estimation. For all CFA and LMI models, covariances were fixed to zero by default. Factor loadings, measurement intercepts, and measurement residuals were constrained stepwise, and configural, metric, and scalar LMI models were compared using model fit criteria, including Akaike’s Information Criterion (AIC), Consistent Akaike’s Information Criterion (CAIC), Bayesian Information Criterion (BIC), and adjusted Bayesian Information Criterion (ABIC), such that lower values reflected better model fit^94^. After the model with the best fit was identified, factor score standardizations (*M* = 0, *SD* = 1) were saved in M*plus* for subsequent analyses. Unlike scale scores or *z*-standardization, factor scores provide partial control over measurement errors while maintaining the core structure of the measurement model^95^.

#### Latent profile analysis

To examine RQ1, we conducted separate latent profile analyses for T_1_ and T_2_, in which profiles were estimated from self- and child-focused emotion regulation strategies. The profile number at each time point was evaluated on the basis of statistical and theoretical criteria. Six latent profile models were freely estimated for each time point and compared using AIC, CAIC, BIC, and ABIC, with lower values indicating better model fit^96^. To determine the optimal number of profiles, we evaluated elbow plots, entropy values, and the Lo-Mendell-Rubin likelihood ratio test (LMR-LRT). Entropy values range from 0 to 1, with higher values suggesting greater classification accuracy. A significant LMR-LRT indicates a significant improvement in the current model over a model with one fewer profile.

#### Latent transition analysis

After determining the number of profiles for T_1_ and T_2_, the final models were integrated for profile similarity by stepwise assessing configural, structural, dispersion, and distributional similarity^43^. These models analyzed similarity over time on the basis of (a) the number of profiles without constraints, (b) constrained intercept indicators, (c) constrained intercept and variance indicators, and (d) constrained profile probabilities. For model comparison, we considered the AIC, CAIC, BIC, and ABIC. The best-fitting model was identified and incorporated into an LTA to address RQ2 by examining the stability of profile membership over time.

RQ3 was investigated by entering the antecedents and outcomes stepwise into the LTA using auxiliary three-step analyses^97,98^, as this M*plus* recommended approach allows predictors and outcomes to be incorporated into the LTA model while correcting for classification error. Mean scores were calculated for all scales. To test predictive similarity, referring to whether the antecedents predicted T_1_ and T_2_ profiles in a comparable way, each antecedent (i.e., gender and educational background) was entered into the final LTA by first freely estimating the variances over time and subsequently constraining the variances. The best-fitting model was selected on the basis of the AIC, CAIC, BIC, and ABIC. Afterwards, all outcomes (parental exhaustion, academic achievement, internalizing problems, and the parent–child relationship) were individually modeled in the final LTA to examine whether the profiles predicted differences in outcomes over time (explanatory similarity). For outcome variables measured at multiple time points (i.e., parental exhaustion, academic achievement), models with freely estimated and constrained variances were compared, and the best-fitting model was determined by the AIC, CAIC, BIC, and ABIC.

## Results

### Descriptive statistics

First, descriptive statistics were investigated. Table 1 provides manifest bivariate correlations for all measures and further presents descriptive statistics, including the mean (*M*), standard deviation (*SD*), minimum, and maximum of the scales. At both time points, the use of self-focused reappraisal (*M*_T1_ = 3.89, *SD*_T1_ = 0.70; *M*_T2_ = 3.83, *SD*_T2_ = 0.73) and child-focused reappraisal (*M*_T1_ = 4.24, *SD*_T1_ = 0.62; *M*_T2_ = 4.18, *SD*_T2_ = 0.63) were reported more frequently than self-focused rumination (*M*_T1_ = 2.46, *SD*_T1_ = 0.92; *M*_T2_ = 2.44, *SD*_T2_ = 0.98) or child-focused rumination (*M*_T1_ = 2.94, *SD*_T1_ = 1.20; *M*_T2_ = 2.91, *SD*_T2_ = 1.13). Construct validity and LMI were confirmed (see the Supplementary Materials, Tables S1 and S2). For all further analyses, factor scores from the scalar LMI model were used.

**Table 1:** Pearson correlation matrix and descriptive statistics of measures

Measure	1.	2.	3.	4.	5.	6.	7.	8.	9.	10.	11.	12.	13.	14.	15.	16.	*M*	*SD*	Min	Max
1. Parental Gender (T_1_)	−																−	−	−	−
2. Parental Education Level (T_1_)	−0.10**	−															3.72	1.23	1.00	7.00
3. Self-focused Reappraisal (T_1_)	−0.01	0.07*	−														3.89	0.70	1.00	5.00
4. Self-focused Rumination (T_1_)	0.14***	−0.07*	−0.16***	−													2.46	0.92	1.00	5.00
5. Child-focused Reappraisal (T_1_)	0.02	−0.03	0.46***	−0.09**	−												4.24	0.62	1.60	5.00
6. Child-focused Rumination (T_1_)	−0.18***	0.05	0.09**	0.08**	0.23***	−											2.94	1.20	1.00	5.00
7. Parental Exhaustion (T_1_)	0.14***	−0.05	−0.22***	0.50***	−0.21***	−0.03	−										1.22	1.35	0.00	6.00
8. Children’s Academic Achievement (T_1_)	−0.06	0.12***	0.04	−0.19***	−0.02	−0.08**	−0.17***	−									10.29	1.84	1.67	13.00
9. Self-focused Reappraisal (T_2_)	−0.04	0.08*	0.59***	−0.16***	0.36***	0.11***	−0.20***	0.04	−								3.83	0.73	1.00	5.00
10. Self-focused Rumination (T_2_)	0.15***	−0.09**	−0.19***	0.66***	−0.10**	0.04	0.46***	−0.18***	−0.19***	−							2.44	0.98	1.00	5.00
11. Child-focused Reappraisal (T_2_)	<0.01	−0.07*	0.42***	−0.07*	0.53***	0.14***	−0.16***	0.03	.46***	−0.13***	−						4.18	0.63	1.20	5.00
12. Child-focused Rumination (T_2_)	−0.21***	0.05	0.07*	0.09**	0.12***	0.58***	−0.03	−0.06	.12***	.04	.24***	−					2.91	1.13	1.00	5.00
13. Parental Exhaustion (T_2_)	0.14***	−0.06	−0.20***	0.42***	−0.19***	−0.06	0.71***	−0.19***	−0.24***	.50***	−0.21***	−0.03	−				1.28	1.41	0.00	6.00
14. Children’s Academic Achievement (T_2_)	−0.06*	0.16***	0.03	−0.18***	0.01	−0.06*	−0.19***	0.65***	.06	−0.22***	.07*	−0.05	−0.24***	−			10.19	1.95	1.00	13.00
15. Children’s Internalization Problems (T_3_)	−0.01	−0.01	−0.16***	0.33***	−0.16***	0.10**	0.32***	−0.24***	−0.12***	.39***	−0.18***	.11***	.39***	−0.30***	−		0.45	0.38	0.00	1.90
16. Parent–Child Relationship (T_3_)	0.08*	−0.06	0.27***	−0.15***	0.33***	−0.06	−0.21***	0.12***	.21***	−0.15***	.33***	−0.03	−0.22***	.18***	−0.35***	−	4.38	0.62	1.00	5.00

T_1_ = time point one; T_2_ = time point two; *p ≤ .05, **p ≤ .01, ***p ≤ .001; *M* = mean; *SD* = standard deviation; Min = minimum; Max = maximum.

### Latent profiles of parents’ self- and child-focused emotion regulation

Based on theoretical considerations and statistical model fit indices, a three-profile model was selected. However, the achieved model fit levels were not entirely consistent across all indices (see Table S3 in the Supplementary Materials for an overview of the fit indices across all profile solutions and time points). Information criteria (AIC, BIC, CAIC, and ABIC) decreased continuously with each additional profile, and the LMR-LRT test remained significant for all six profiles at T_1_ and up to four profiles at T_2_. However, elbow plots suggested bends at three and four profiles across both time points. Additionally, the three-profile solution was strongly supported by (a) the fourth profile demonstrating limited qualitative distinctiveness and interpretive value, as evidenced by marginal mean differences compared to the first profile across three of the four assessed strategies, (b) an overidentified four-profile model in the LTA, indicating a model misspecification, and (c) a high-class accuracy for the three-profile model (Entropy_T1_ = 0.82, Entropy_T2_ = 0.83). Thus, a three-profile model was selected, and subsequently, LMI was tested. All further analyses were based on the scalar model, as it showed the best model fit (see Table S4 in the Supplementary Materials), indicating invariance in the profile structures, sample sizes, and means. We carefully assessed profile distinctiveness through visual inspection and by re-estimating the solution with multiple random starts^99^, and found no indication of the presence of a Salsa effect in our data (i.e., classes reflect mainly quantitative differences in the indicators).

**Fig. 1:**
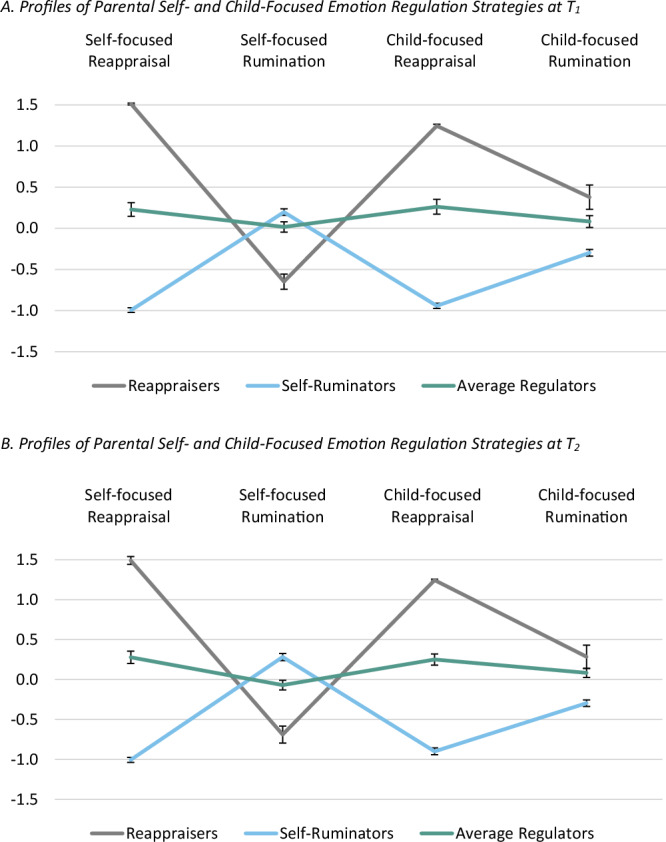
Profiles of parental self- and child-focused emotion regulation at T_1_ and T_2_. Note. T_1_ = time point one; T_2_ = time point two; each emotion regulation strategy in the profiles is reported with error bars representing ±1 standard error of the mean; *N* = 1046 participants.

The three profiles showed visually similar structural patterns across the two time points (see Fig. 1). The first profile was labeled *reappraisers*, due to the highest levels of self- and child-focused reappraisal relative to all other profiles and the sample averages. Interestingly, reported levels of child-focused rumination were elevated in comparison with the other profiles and the averages of the sample, whereas self-focused rumination levels were relatively low. Conversely, individuals in the second profile showed relatively low levels of self- and child-focused reappraisal, along with reduced child-focused rumination. This profile was characterized by the highest levels of self-focused rumination across all profiles, leading to the label *self-ruminators*. Lastly, the third profile, labeled *average regulators*, was characterized by mid-range scores across all emotion regulation strategies with slightly elevated levels of self- and child-focused reappraisal compared with the sample’s averages, which were still significantly lower than in the *reappraisers* profile. In Table 2, we report profile demographics for the emotion regulation strategies included in the profiles and the profile correlates. Differences between profiles were tested using analyses of variance (ANOVAs) for continuous variables and chi-square tests for categorical variables.

**Table 2:** Profile demographics at T_1_ and T_2_

A. Profile Demographics T_1_					
	Reappraisers T_1_ (*n* = 91)	Self-Ruminators T_1_ (*n* = 264)	Average Regulators T_1_ (*n* = 692)	Test statistic	*p*-Value
Self-focused Reappraisal T_1_ (*M*, *SE*)	1.51 (0.01)	−0.10 (0.08)	0.23 (0.03)	*F*(2, 1043) = 873.40	<0.001
Self-focused Rumination T_1_ (*M*, *SE*)	−0.65 (0.01)	0.20 (0.06)	0.02 (0.04)	*F*(2, 1043) = 31.14	<0.001
Child-focused Reappraisal T_1_ (*M*, *SE*)	1.25 (0.02)	−0.94 (0.09)	0.26 (0.03)	*F*(2, 1043) = 549.90	<0.001
Child-focused Rumination T1 (M, SE)	0.38 (0.15)	−0.30 (0.07)	0.08 (0.04)	*F*(2, 1043) = 20.38	<0.001
Parental Gender T_1_ (*n*, % per profile)				χ^2^(2) = 1.67	.433
Male	50 (54.95%)	110 (47.83%)	347 (47.86%)		
Female	41 (45.05%)	120 (52.17%)	378 (52.14%)		
Parental Educational Level T_1_ (*n*, % per profile)				χ^2^(4) = 2.50	.500
Lower than Secondary School	2 (2.20%)	4 (1.74%)	10 (1.38%)		
Secondary School	13 (14.29%)	49 (21.30%)	153 (21.10%)		
Higher Education Degree*	75 (82.42%)	174 (75.65%)	539 (74.34%)		
Other	1 (1.10%)	3 (1.30%)	23 (3.17%)		
Parental Exhaustion T_1_ (*M*, *SE*)	0.34 (0.07)	1.66 (0.10)	1.19 (0.05)	*F*(2, 1043) = 33.97	<0.001
Children’s Academic Achievement T_1_ (*M*, *SE*)	10.33 (0.21)	10.25 (0.13)	10.30 (0.07)	*F*(2, 1026) = 0.08	.920
Children’s Internalizing Problems T_3_	0.26 (0.03)	0.53 (0.03)	0.44 (0.02)	*F*(2, 872) = 14.67	<0.001
Parent–Child relationship T_3_	4.75 (0.06)	4.07 (0.05)	4.43 (0.02)	*F*(2, 872) = 43.10	<0.001

B. Profile Demographics T_2_					
	Reappraisers T_2_ (*n* = 78)	Self-Ruminators T_2_ (*n* = 326)	Average Regulators T_2_ (*n* = 643)	Test statistic	*p*-Value
Self-focused Reappraisal T_2_ (*M*, *SE*)	1.49 (0.05)	−1.01 (0.08)	0.28 (0.03)	*F*(2, 1043) = 943.60	<0.001
Self-focused Rumination T_2_ (*M*, *SE)*	−0.70 (0.11)	0.28 (0.06)	−0.07 (0.05)	*F*(2, 1043) = 37.00	<0.001
Child-focused Reappraisal T_2_ (*M*, *SE*)	1.24 (0.01)	−0.90 (0.07)	0.25 (0.04)	*F*(2, 1043) = 516.00	<0.001
Child-focused Rumination T_2_ (*M*, *SE*)	0.29 (0.15)	−0.30 (0.06)	0.08 (0.04)	*F*(2, 1043) = 22.23	<0.001
Parental Exhaustion T_2_ (*M*, *SE*)	0.57 (0.10)	1.81 (0.10)	1.14 (0.05)	*F*(2, 1043) = 35.95	<0.001
Children’s Academic Achievement T_2_ (*M*, *SE*)	10.60 (0.19)	10.00 (0.13)	10.23 (0.07)	*F*(2, 1029) = 3.27	0.038

*N* = 1,046 participants; T_1_ = time point one; T_2_ = time point two; T_3_ = time point three; * Higher Education Degree is summarized from the categories Associate’s, Bachelor’s, Master’s, and PhD, which were each entered separately in the analyses.

### Profile stability over time

**Fig. 2:**
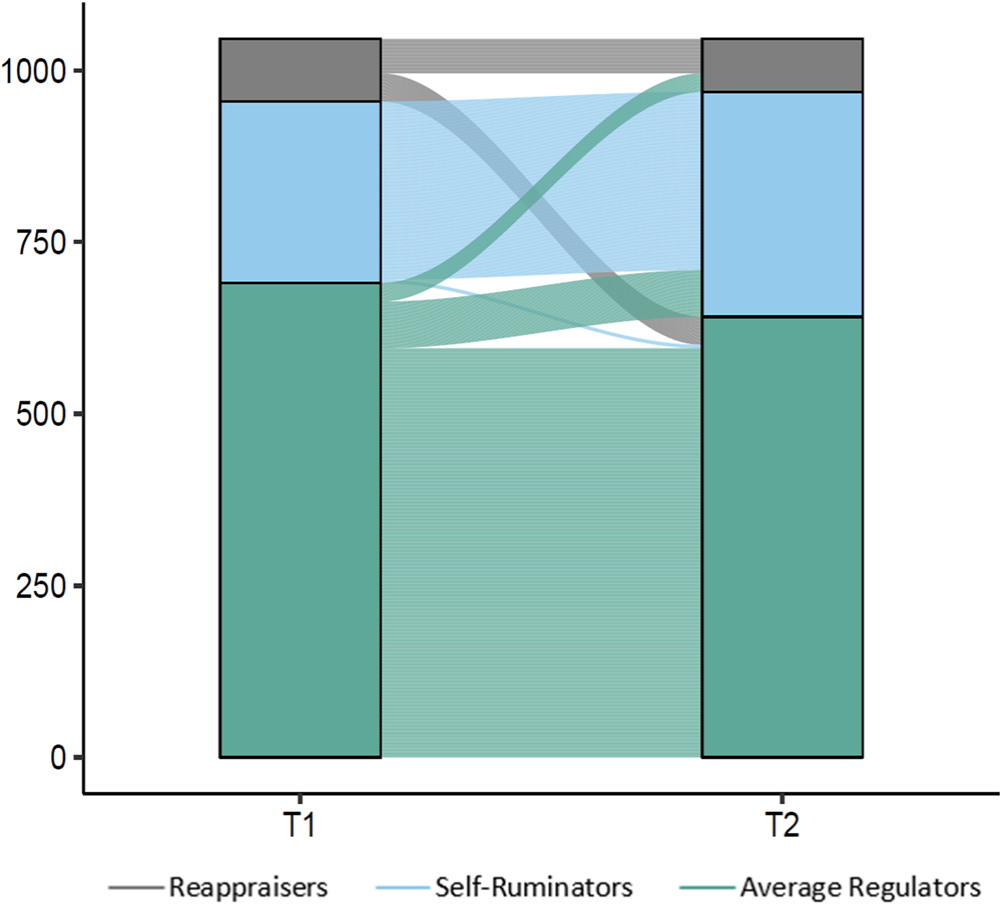
Transitions of profiles from T_1_ to T_2_. Note. T_1_ = time point one; T_2_ = time point two; the x-axis represents the two time points, while the y-axis indicates the total number of participants (*N* = 1046 participants).

Latent transition scores from T_1_ to T_2_ were computed (see Fig. 2 and Table 3). *Reappraisers* demonstrated moderate profile membership stability, with approximately 55% of individuals remaining in this profile at T_2_. A substantial proportion ( ≈ 45%) transitioned to *average regulators*, whereas transitions to *self-ruminators* were rare. The most stable profile was *self-ruminators*, with approximately 98% of individuals remaining in this profile across time points, indicating minimal profile change. *Average regulators* also showed high stability ( ≈ 86%), though a small percentage transitioned to *reappraisers* (≈ 4%) or *self-ruminators* (≈ 10%). Based on the estimated model, the number of individuals in the *reappraisers* and *average regulators* profiles decreased from T_1_ to T_2_ (*reappraisers*: *n*_T1_ ≈ 90, *n*_T2_ ≈ 77; *average regulators*: *n*_T1_ ≈ 692, *n*_T2_ ≈ 643), whereas the number of individuals in the *self-ruminators* profile increased over time (*n*_T1_ ≈ 263, *n*_T2_ ≈ 326).

**Table 3:** Latent transition probabilities from T_1_ to T_2_ (in percent)

Profiles	Reappraisers (T_2_)	Self-Ruminators (T_2_)	Average Regulators (T_2_)
Reappraisers (T_1_)	55.2%	<1.0%	44.8%
Self-Ruminators (T_1_)	<1.0%	98.3%	1.7%
Average Regulators (T_1_)	4.0%	9.8%	86.2%

T_1_ = time point one; T_2_ = time point two; *N* = 1046 participants.

### Antecedents and outcomes of parental emotion regulation profiles

In the next step, we investigated whether parental gender, and educational level predicted profile membership (see Table 4). Across all antecedents, more constrained models consistently provided the best model fit. Parental gender and educational level were not significantly different across the three profiles. Afterwards, we incorporated the outcomes parental exhaustion (T_1_ and T_2_), children's academic achievement (T_1_ and T_2_), children’s internalizing problems (T_3_), and the parent–child relationship (T_3_) into our model to examine whether they differed by profile membership (see Table 5). For models that included outcomes measured at multiple time points (e.g., children’s academic achievement at T_1_, children’s academic achievement at T_2_), more constrained models demonstrated superior fit. Results highlighted opposing patterns for *reappraisers* and *self-ruminators*, with *average regulators* showing intermediate levels in contrast to the other two profiles for parental exhaustion, children’s internalizing problems, and the parent–child relationship. *Reappraisers* reported the lowest levels of parental exhaustion and children’s internalizing problems, as well as the highest quality of parent–child relationships. By contrast, *self-ruminators* showed the highest levels of parental exhaustion and children’s internalizing problems, accompanied by the poorest quality of parent–child relationships. Children’s academic achievement did not differ significantly across the three profiles in the LTA.

**Table 4:** Relationships between profile membership and antecedents

	Reappraisers vs. Self-Ruminators		Reappraisers vs. Average Regulators		Self-Ruminators vs. Average Regulators	
Antecedent	β/SE	OR [CI]	β/SE	OR [CI]	β/SE	OR [CI]
Gender	−0.30/0.21	0.74 [0.49, 1.12]	−0.25/0.18	0.78 [0.55, 1.11]	0.05/0.14	1.05 [0.79, 1.39]
Educational Level	0.08/0.09	1.08 [0.92, 1.28]	0.02/0.07	1.02 [0.89, 1.18]	−0.06/0.06	0.94 [0.84, 1.06]

β = coefficient; SE = standard error of coefficient; OR = odds ratio; CI = 95% confidence interval; *p ≤ 0.05, **p ≤ 0.01, ***p ≤ 0.001; *N* = 1046 participants.

**Table 5:** Relationships between profile membership and outcomes

	Reappraisers (REAP)	Self-Ruminators (RUMN)	Average Regulators (AVRG)	
Outcome	*M* [CI]	*M* [CI]	*M* [CI]	Significant differences between profiles
Parental Exhaustion T_1_ & T_2_	0.43 [0.31, 0.56]	2.01 [1.74, 2.28]	1.01 [0.88, 1.13]	RUMN > AVRG > REAP
Academic Achievement T_1_ & T_2_	10.44 [10.15, 10.75]	10.07 [9.83, 10.30]	10.30 [10.18, 10.41]	REAP = RUMN = AVRG
Child Internalizing Problems T_3_	0.31 [0.25, 0.36]	0.55 [0.51, 0.60]	0.41 [0.38, 0.44]	RUMN > AVRG > REAP
Parent–Child Relationship T_3_	4.80 [4.72, 4.88]	4.04 [3.95, 4.13]	4.49 [4.45, 4.53]	REAP > AVRG > RUMN

*M* = mean; CI = 95% confidence interval; T_1_ = time point one; T_2_ = time point two; T_3_ = time point three; *N* = 1046 participants.

## Discussion

With this study, we aimed to identify profiles of parental self- and child-focused reappraisal and rumination (RQ1) and examine their stability (RQ2). With RQ3, we tested the profiles’ associations with antecedents (i.e., parental educational level and gender) and outcomes (i.e., parental exhaustion, children’s internalizing problems, children’s academic achievement, and the parent–child relationship). We identified three distinct emotion regulation profiles, labeled *reappraisers* (T_1_ ≈ 9%, T_2_ ≈ 7%), *self-ruminators* (T_1_ ≈ 25%, T_2_ ≈ 31%), and *average regulators* (T_1_ ≈ 66%, T_2_ ≈ 61%). All profiles showed moderate to high stability across 4 months, with self-ruminators demonstrating the highest stability (*reappraisers* ≈ 55%, *self-ruminators* ≈ 98%, *average regulators* ≈ 86%). Contrary to our hypotheses, parents’ educational level and gender did not significantly predict profile membership. Results from the transition analyses showed significant profile differences in all outcomes except children’s academic achievement. Specifically, *reappraisers* reported the lowest levels of parental exhaustion (T_1_ and T_2_) and children’s internalizing problems (T_3_) as well as the highest levels of parent–child relationship closeness (T_3_). By contrast, *self-ruminators* reported the highest levels of parental exhaustion (T_1_ and T_2_) and children’s internalizing problems (T_3_) as well as the lowest levels of parent–child relationship closeness (T_3_). *Average regulators* showed intermediate levels on these outcomes, positioned between the other two profiles.

Our results revealed three profiles, *reappraisers, self-ruminators*, and *average regulators*, which partly aligned with our expectations regarding profile constitution. Parents who reported higher levels of self-focused reappraisal also reported higher levels of child-focused reappraisal, consistent with prior research demonstrating congruence in the emotion regulation strategies parents employ for themselves and their children^44^. In comparison, self- and child-focused rumination were reported at much lower levels and did not show aligning patterns. Self-focused rumination showed patterns opposite to self-focused reappraisal in all profiles, in line with prior literature reporting divergent associations of the two strategies regarding outcomes such as well-being^6^. Contrary to our hypotheses, child-focused rumination followed a similar pattern as child-focused reappraisal across profiles, consistent with some prior variable-focused analyses showing positive associations between child-focused reappraisal and rumination^46^. These patterns could indicate conceptual differences in self- and child-focused rumination, as self-focused rumination typically involves downward spiraling in solitude, whereas child-focused rumination occurs in a social context. Such differences are also reflected in the respective measurement scales, where self-focused rumination typically includes reflection and brooding subscales, which capture negative self-focus and self-blame as the two key mechanisms underlying rumination^4,5^. Child-focused rumination, on the other hand, is assessed through reflection only, which potentially attenuates the role of self-focus^4,82^. Reappraisal and rumination might also be more closely related in interpersonal contexts, as the reflective aspect of child-focused rumination could represent parents’ attempts to encourage their children to think through their experiences as well as increased involvement. Future research should examine the robustness of our results to better understand similarities and differences in the composition of reappraisal and rumination in self- and child-focused contexts.

Supporting our hypotheses, all three profiles showed moderate to high stability over the 4-month period. *Reappraisers* demonstrated moderate stability (55%), with most transitions occurring towards the *average regulators* profile. *Self-ruminators* demonstrated the highest stability, with approximately 98% of parents remaining in this profile after 4 months. Since the study investigated habitual emotion regulation strategy use, moderate stability was expected and confirmed the robustness of the measurement tools. However, results from our transition analyses showed differential stability of the profiles and highlighted the concerning persistence of rumination over time, which has relevant implications for clinical practice especially given that increased self-rumination, but not decreased self-focused reappraisal, has been observed as a stable feature of individuals with a history of depression^4,100^. *Average regulators* were also highly stable (86%), with only a few individuals transitioning to the other two profiles. Nevertheless, the transitions of the profiles could also indicate changes in the need to regulate emotions in response to increasing or decreasing external and internal demands.

The three profiles differed substantially in size with the *average regulators* profile being the largest profile (*n*_T1_ ≈ 692; *n*_T2_ ≈ 643), and the *reappraisers* profile being the smallest (*n*_T1_ ≈ 90; *n*_T2_ ≈ 77). When interpreting small groups, such as the *reappraisers* profile, it is important to consider their qualitative contribution, as estimates for small profiles can be less precise and statistical power can be reduced^101^. However, the *reappraisers* profile provided valuable insight into a potentially highly functional emotion regulation pattern and did not fall below common minimum-size guidelines (i.e., 5% of the sample or 50 cases)^102^. The *self-ruminators* profile represented the second largest group and was the only one to increase in size over time (*n*_T1_ ≈ 263; *n*_T2_ ≈ 326). These results may reflected rising stress and regulatory demands as the school year progresses, given that T_1_ occurred at the start of children’s school year. Interestingly, the overall sample mean for self-focused rumination remained stable across both time points, whereas the *self-ruminators* showed a slight increase in self-focused rumination, demonstrating a central advantage of person-centered approaches in identifying subgroup-specific changes that are not captured by aggregated means. The unequal profile sizes might partly also reflect the limited number of assessed emotion regulation strategies, as different profiles may have emerged if additional strategies had been included. Still, our findings underscored the need for tailored interventions that specifically target rumination to counteract its negative effects.

As expected, the profiles significantly predicted parental exhaustion (T_1_ and T_2_), and examining this association provided deeper insight into the meaning of the profiles themselves. Parents in the *self-ruminators* profile showed the highest levels of parental exhaustion, followed by *average regulators* and, lastly, *reappraisers*. As the name suggests, *self-ruminators* had the highest levels of self-focused rumination compared with the other profiles, but they also reported very low levels of the three other strategies, particularly self- and child-focused reappraisal. This profile may have included parents who are highly exhausted and engaged in frequent self-focused rumination, leaving them with limited capacities to regulate their emotions using potentially helpful strategies (e.g., self-focused reappraisal). Associated with this exhaustion, parents in this profile may have been absorbed by their own emotions, which could have reduced their ability to focus on their children’s needs^103^ and explain the low levels of child-focused emotion regulation in this profile, potentially indicating some disengagement from parenting practices. Alternatively, these parents might have recognized the maladaptive nature of self-focused rumination and actively tried not to transfer it to their children by avoiding child-focused rumination.

By contrast, the *reappraisers* can be considered the most high-functioning group of parents with respect to parental exhaustion. They appeared to know which strategies are helpful for regulating their own emotions and displayed flexibility in using strategies to support their child’s regulation. Nevertheless, parents’ habitual patterns of self-focused reappraisal and rumination are bidirectionally associated with parents’ healthy functioning^24,52,72^. Therefore, it is important to consider that our measured correlates may also have contributed to the profiles. For example, higher child internalizing problems or greater parental exhaustion might have increased parents’ self-focused rumination, whereas in the *reappraisers* profile, lower parental exhaustion and fewer child internalizing problems might have been linked to parents’ greater capacity to use self- and child-focused reappraisal^38^. Similarly, it has been posited that parents experience lower distress when their child becomes better regulated^72^. Although all three profiles were significantly different in parental exhaustion, the overall exhaustion levels were relatively low in our sample (*M*_T1_ = 1.22, *SD*_T1_ = 1.35; *M*_T2_ = 1.28, *SD*_T2_ = 1.41). Future research should investigate these profiles in clinical populations with a larger proportion of parents experiencing parental exhaustion or other symptomatology.

Contrary to our expectations, parental gender and educational level at T_1_ did not predict profile membership. However, our sample was skewed towards parents with higher educational levels (62.91% with a bachelor’s degree or higher). Previous research indicated that interventions and emotion regulation strategy use were particularly beneficial for individuals with lower SES^104^, potentially due to limited buffering resources (e.g., access to therapy services or childcare support), whose underrepresentation in our sample might have contributed to the results. A notable strength of this study is the balanced sample of mothers and fathers, with 48.47% of participants being fathers, a group that is often scarce in developmental psychology research. Gender differences are frequently reported for self-focused rumination^4,48^, with females typically showing higher levels of self-focused rumination. However, prior findings on child-focused rumination suggested higher levels in fathers^44,46^, and the literature on self- and child-focused reappraisal in relation to gender differences remains mixed, with some evidence indicating no gender differences in self-focused reappraisal and emotion socialization^3,54,58^. These diverging findings regarding the four assessed strategies may partly explain why no gender differences emerged between profiles. Future research should investigate socioeconomic variables and interactions between parental and child gender more closely to better understand potential differential effects across sociodemographic groups.

As hypothesized, our results showed that the three profiles differed significantly in children’s internalizing problems at T_3_, although the reported levels were relatively low (*M*_T3_ = 0.45; *SD*_T3_ = 0.38). Parents in the *self-ruminators* profile reported the highest levels of children’s internalizing problems, followed by *average regulators* and *reappraisers*. These findings suggested that the *reappraisers* profile was, beyond parental exhaustion, also the most adaptive one regarding children’s internalizing problems. Considering the profile characteristics, self-focused emotion regulation may have had stronger associations with children’s emotional outcomes, as witnessing parental distress may have weighed more heavily on children than the specific child-focused emotion regulation strategies used, especially when parental symptoms reach a clinical cutoff. Avoiding child-focused rumination might not have been protective if parents engaged in self-focused rumination, as this discrepancy may have appeared inauthentic or reduced the effectiveness of their child-focused emotion regulation implementation. Conversely, parents in the *reappraisers* profile might have experienced higher well-being, enabling them to be less preoccupied with their own difficulties and more attuned to their children’s needs^103^. Thus, our findings highlighted the significant role of parental self-focused emotion regulation for family well-being and potential spillover effects of parental well-being to their children’s internalizing problems.

Unexpectedly, the transition analyses did not predict children’s academic achievement at T_1_ or T_2_, a finding that could have demonstrated the complexity and relative distance of academic achievement as an outcome of parental emotion regulation, especially compared with affective and relationship-related outcomes. Interestingly, the profiles differed significantly in academic achievement at T_2_, with *reappraisers* reporting the highest achievement and *self-ruminators* the lowest. However, academic achievement showed low variability in our sample, with high achievement in children being overrepresented (*M*_T1_ = 10.29, *SD*_T1_ = 1.84; *M*_T2_ = 10.19, *SD*_T2_ = 1.95). Thus, the findings of the transition analyses may reflected lower stress in achievement-related situations among children in this sample, reducing the need for parental regulation. Also, children’s self-focused emotion regulation can relate to academic achievement^105–107^, but evidence of parental child-focused emotion regulation remains limited. We focused on child-focused emotion regulation use in the context of achievement emotions, and future work should consider moderators, such as parents’ ability to detect their children’s regulatory needs and effectiveness^42,103^. Additionally, existing evidence offers mixed conclusions about the effectiveness of reappraisal in regulating achievement-related emotions^46,107,108^, with strategies such as problem-solving and competence development potentially being of relevance. Hence, such strategies should be considered in future research.

Comparable to parental exhaustion and children’s internalizing problems, the parent–child relationship (T_3_) differed significantly across profiles, with *reappraisers* reporting the highest relationship closeness, *average regulators* reporting intermediate levels, and *self-ruminators* reporting the lowest. Overall, it is important to note that the reported relationship closeness was relatively high in the whole sample (*M*_T3_ = 4.39, *SD*_T3_ = 0.62). Nevertheless, child-focused rumination was elevated in the more adaptive profile with respect to the parent–child relationship, and parents’ self-focused rumination was not. One possibility for these results is that child-focused reappraisal compensated for the potential negative effects of child-focused rumination. Furthermore, a closer parent–child relationship might have involved more frequent interactions, which could also have included child-focused rumination. Accordingly, the use of child-focused emotion regulation might have reflected parental attempts to assist their child with negative emotions, which in itself could have demonstrated engagement and care. Maintaining contact and communication with the child may have been the critical factor for the parent–child relationship, even if regulation strategies were not always optimally chosen. This could have also explained why the *self-ruminators* profile, where both child-focused emotion regulation strategies were lowest compared to the other profiles, was associated with the lowest relationship closeness. In Grades 6–9, the parent–child relationship becomes more egalitarian and bidirectional^24,26^, with a more distant relationship potentially also eliciting less parental regulation.

In sum, the current study offered several theoretical and practical implications, particularly for parenting emotion regulation frameworks. The identified profiles indicated the importance of parents’ self-focused emotion regulation, which is noteworthy given that our sample included parents of adolescents in Grades 6–9, a developmental period characterized by increased autonomy^26,27^. These findings highlighted the continued importance of parental modeling as a mechanism across development while also suggesting that parenting behaviors may exert comparably less direct relations as children become more autonomous. The profiles’ characteristics also provided evidence of the application of a “good-enough” parenting model to emotion regulation, in which high-functioning parents may combine effective self-regulation with flexible, sometimes imperfect, approaches to child regulation. Our findings also indicated that results from self-focused emotion regulation cannot be automatically generalized to child-focused contexts, which is why interpersonal contexts need to be given more attention in future emotion regulation research. Practically, our results highlighted the importance of preventions to decrease the risk of more severe mental health conditions in parents (e.g., parental burnout) and their spillover and reciprocal effects on child development^67,71^. Lastly, the results underscored the relevance of social emotion regulation dynamics between parents and children, which may play a preventive role during adolescence, a vulnerable period for the development of psychopathology^4,32^. Thus, our findings can serve as a foundation for future developmental and clinical research aimed at promoting parental well-being and adolescent adjustment.

### Limitations

This study has several limitations that should be considered when interpreting the findings and deriving directions for future research. Although the person-centered approach is well-suited for identifying subgroups, LPA and LTA assume that the observed distribution in the sample reflects the distribution of underlying populations, reduce variances as groups are estimated to maximize distinctiveness, and the methodological process includes subjective decisions regarding the final models^99,102,109^. Additionally, we relied on self-report questionnaires from one parent per family to feasibly recruit a large sample, but self-reports are subject to bias and social desirability effects^110^. The high scores observed for parent–child relationship closeness and academic achievement also suggest potential reporting bias, and relying on one parent’s perspective captures only a partial view of the family dynamics. For child-focused emotion regulation, we adapted the items to academic achievement contexts, which may have introduced limitations such as a potential misalignment between self- and child-focused emotion regulation that could have influenced profile membership and the profiles’ associations with antecedents and outcomes. Some research suggested that strategy effectiveness differs between academic and non-academic contexts^42^, which could imply that parents’ child-focused emotion regulation strategy use also varies by context. Therefore, future studies should directly compare child-focused strategy use across academic and non-academic situations. Additionally, only two strategies (i.e., reappraisal and rumination) were considered for self- and child-focused emotion regulation. Future research should include a broader range of strategies, including problem-solving and avoidance, which have recently been shown to be relevant for achievement regulation^107^. Finally, the study included constraints on generality, as the data were collected in a single Western country (US), and cultural differences may limit the generalizability of the findings to other populations^111^. Also, the sample had a higher educational background than the general population, with 62.91% of parents holding a university degree, and parents’ gender was measured as a binary variable. Future studies should address these limitations by recruiting more diverse samples from different cultural contexts.

## Conclusion

Taken together, our findings highlighted the close connections of parental self- and child-focused emotion regulation patterns with family well-being and parent–child relationship closeness. Identifying distinct profiles of reappraisal and rumination showed that parents who more frequently used self-focused rumination were at greater risk of parental exhaustion, children’s internalizing problems, and lower relational closeness compared with parents who frequently used self- and child-focused reappraisal. Although child-focused emotion regulation was adapted to parental regulation of achievement emotions, children’s academic achievement was not associated with longitudinal profiles of parental emotion regulation. Still, our research demonstrated that during adolescence, parental emotion regulation remained closely associated with the parent–child relationship as well as parent and child well-being, and emphasized the importance of tailored interventions for distinct subgroups.

## Supplementary information

Transparent Peer Review file

Supplementary Materials

## Data Availability

The project’s preregistration can be accessed on the OSF at https://osf.io/4hvtj, and the data sets can be accessed on the OSF at https://osf.io/ybt46/.

## Code availability

The project’s R codes and M*plus* codes are publicly available on OSF at https://osf.io/ybt46/. The project DOI is 10.17605/OSF.IO/YBT46.
