# Supplementary material for: Longitudinal profiles of parental self- and child-focused emotion regulation
Source: Commun Psychol. 2026 May 16;4:115. doi: 10.1038/s44271-026-00469-w (PMC13469115; doi:10.1038/s44271-026-00469-w)
Supplement: Supplementary file 1 [file 44271_2026_469_MOESM1_ESM.pdf]

nature portfolio

Peer Review File

Longitudinal Profiles of Parental Self- and Child-Focused Emotion Regulation

Corresponding Author: Ms Gesine Jordan

This file contains all editorial decision letters in order by version, followed by all author rebuttals in order by version.

Version 0:

Decision Letter:

** Please ensure you delete the link to your author homepage in this e-mail if you wish to forward it to your coauthors **

Dear Ms Jordan,

Thank you for your patience during the peer-review process. Your manuscript titled "Profiles of Parental Self- and Child-Focused Emotion Regulation: Longitudinal Associations with Parental Exhaustion, Children's Internalizing Problems, Children's Achievement, and the Parent–Child Relationship" has now been seen by 2 reviewers, and I include their comments at the end of this message. They find your work of interest but raised some important points. We are interested in the possibility of publishing your study in Communications Psychology, but would like to consider your responses to these concerns and assess a revised manuscript before we make a final decision on publication.

We therefore invite you to revise and resubmit your manuscript, along with a point-by-point response to the reviewers. Please highlight all changes in the manuscript text file.

Editorially, we consider it important that you address the theoretical, methodological, and analytic concerns of the reviewers. We request that you include the relevant background literature and address the reviewers' concerns about potential effects influencing your analyses. The reviewers have provided recommendations for streamlining the introduction. Please use care to avoid causal language where no causal evidence is available.

Please note that we consider it crucial that you can demonstrate that the profiles are separate (i.e., that they are not the result of the salsa effect). Without this, we won't be able to consider a revised version as we consider this a fundamental aspect of the advance that makes the work potentially suitable for publication in the journal.

As you revise the manuscript in response to these issues, please also implement all requests in the attached Mandatory Revision Requests document. All requirements listed in this document need to be fully met, or the work will be returned to you for further revisions without peer review. This workflow is in place to increase the likelihood that the paper will be accepted for publication. It reduces the number of rounds of revision (and review) and ensures that the reviewers vet a version of the article that is compliant with journal policies. If you have any questions regarding the required revisions, please contact the journal prior to resubmission to avoid a negative outcome.

We are committed to providing a fair and constructive peer-review process. Please don't hesitate to contact us if you wish to discuss the revision in more detail.

Please submit the following items:

- Revised manuscript
- Point-by-point response to the referees' comments
- Mandatory Revision Requests Table (attached).
- Cover letter (as a separate document)

via this link: Link Redacted .

** This url links to your confidential home page and associated information about manuscripts you may have submitted or are reviewing for us. If you wish to forward this email to co-authors, please delete the link to your homepage first **

We hope to receive your revised paper within 8 weeks; please let us know if you aren't able to submit it within this time so that we can discuss how best to proceed. If we don't hear from you, and the revision process takes significantly longer, we may close your file. In this event, we will still be happy to reconsider your paper at a later date, provided it still presents a significant contribution to the literature at that stage.

We look forward to seeing the revised manuscript and thank you for the opportunity to review your work.

Best regards,

Nida Ali

Nida Ali, PhD
Editorial Board Member
Communications Psychology
orcid.org/0000-0003-4921-7940

REVIEWER EXPERTISE:
longitudinal research, latent profile analyses and latent transition analyses, parent- child relationships

REVIEWER REPORTS:

Reviewer #1 (Remarks to the Author):

The authors present a latent class profile analysis from two timepoints using a dataset on emotional regulation responses (reappraisal and rumination) and their association with parental exhaustion, children's Internalizing Problems, children's achievement. The article is well written and the topic deserves attention. Other strengths include the second data point and sample size. I think the introduction could benefit from more structure. The latent class profile analysis might reflect the Salsa effect which would point to the lack of different profiles in the population. I have attached my comments below and hope the authors find them helpful. Please note that my perception of the manuscript is subjective and my feedback includes only suggestions. I wish the authors the best of luck and success with their research.

Signed
Aljoscha Dreisoerner

Introduction
• Overall, I found the introduction far too long and as a result a bit disjointed and unfocused. You introduce a lot of concepts and discuss their antecedents and outcomes, but in my reading, the main contribution of the current study becomes harder to identify that way
• In addition, I am not sure the introduction reflects a full account of the studies describ ng co-regulation strategy use of parents and their children, which I understand is the main focus of the current study. What does the current literature say on how parents regulate themselves and their children's emotions at the same time?
• The writing in the section "Self- and Child-Focused Emotion Regulation" is a bit disjointed (particularly in the first paragraph)
• I am not sure I would call rumination a strategy without first introducing the difference between adaptive and maladaptive coping. Rumination is a symptom in many disorders and referring to it as a strategy without this qualification reads like people choose to ruminate, which they most likely do subconsciously.

Results
• One key Table isn't included: What are the descriptive statistics of important variables (outcomes, antecedents) by latent profile class. See https://doi.org/10.1038/s41398-021-01508-2 Table 3 for an example (please also include rumination and reappraisal)
• Please carefully check if the solution does not reflect the Salsa effect, which just forces a single population into separate latent classes on one outcome variable. I could not verify this from the existing tables
https://pmc.ncbi.nlm.nih.gov/articles/PMC7746621/
• I now see Figure 1 and I do have the suspicion that your solution represents the Salsa effect that divided groups into high reappraisal, moderate reappraisal and low reappraisal. Since reappraisal and rumination are strongly negatively correlated too you might have gotten the current outcome. Please discuss. If the Salsa effect is present, a variable oriented approach

like multi-level modeling might be more suited to the current data or could at least complement the current analysis as a robustness check.
• The labels for the bars in Figure 1, make the graph hard to understand
• Tables dont explain abbreviations currently
• I would like to see a table that lists key variables by latent profile class (also demographics)
• Maybe I missed it, but what was the dropout in this study?

Discussion
• In latent class analysis, it can become an issue when one class is particularly small. Please discuss the methodological and theoretical implications of this some more.
• The limitations currently dont address the inherent limitations of latent class analysis

Minor
• For readability, I would recommend not abbreviating emotional regulation

Reviewer #2 (Remarks to the Author):

Thank you for the opportunity to review this manuscript, which presents interesting findings that contribute to the literature on intra and interpersonal emotion regulation. I think that the method and results sections are overall well-written and clear, but the introduction and discussion sections have room for improvement. A number of my comments revolve around (1) authors implying that there is no or limited literature on topics that are well-researched and (2) authors' use of causal language. I hope that authors find these revisions helpful as they move forward this manuscript.

Abstract

Authors write that patterns of relationships between the variables they focus on remain "unexplored"—a term such as "underexplored" would be better, though that also is not quite accurate because there are large literatures on relations between the variables studied in this paper. See below for a few examples that authors do not cite in the paper (critical reviews that authors do helpfully cite are Zimmer-Gembeck et al., 2022; Zitzmann et al., 2024; and Brandao et al., 2024). I don't think that these need to be cited in the abstract specifically, but citing somewhere the four reviews/meta-analyses below on parenting ER frameworks would help support the current study.

Crandall, A., Deater-Deckard, K., & Riley, A. W. (2015). Maternal emotion and cognitive control capacities and parenting: A conceptual framework. Developmental Review, 36, 105–126. https://doi.org/10.1016/j.dr.2015.01.004

Hajal, N. J., & Paley, B. (2020). Parental emotion and emotion regulation: A critical target of study for research and intervention to promote child emotion socialization. Developmental Psychology, 56(3), 403–417. https://doi.org/10.1037/dev0000864

Zhang, X., Beatty, A., Abela, K., Fernandes Melo, M., Kenny, M., Atkinson, L., & Gonzalez, A. (2023). Assessing parental emotion regulation in the context of parenting: A systematic review. Developmental Review, 69, 1–37. https://doi.org/10.1016/j.dr.2023.101092

Edler, K., & Valentino, K. (2024). Parental self-regulation and engagement in emotion socialization: A systematic review. Psychological Bulletin, 150(2), 154–191. https://doi.org/10.1037/bul0000423

Compas, B. E., Jaser, S. S., Bettis, A. H., Watson, K. H., Gruhn, M. A., Dunbar, J. P., Williams, E., & Thigpen, J. C. (2017). Coping, emotion regulation, and psychopathology in childhood and adolescence: A meta-analysis and narrative review. Psychological Bulletin, 143(9), 939–991. https://doi.org/10.1037/bul0000110

Intro

On p.3, authors write that "longitudinal patterns of parental self- and child-focused ER have not been explored." Child-focused ER, as the authors describe, is very similar to the concept of parental emotion socialization (efforts to change children's emotions via parents' responses to children's emotions). Edler & Valentino (2024) summarize literature on parental ER and parental emotion socialization behaviors, and cover several articles that present longitudinal associations between parental ER and parental supportive and nonsupportive reactions to children's emotions: for example, see Price & Kiel (2022). Although I understand that authors may mean that few (or no) studies have conducted profile analyses with the specific variables they use, and examined whether these profiles change over time, this literature is important to acknowledge—as currently written, the statement "longitudinal patterns of parental self- and child-focused ER have not been explored" is misleading.

Price, N. N., & Kiel, E. J. (2022). Longitudinal links among mother and child emotion regulation, maternal emotion socialization, and child anxiety. Research on Child and Adolescent Psychopathology, 50(2), 241–254. https://doi.org/10.1007/s10802-021-00804-1

On p.3, authors refer to "parental profiles" without explaining what they mean by profiles. Later, they explain they will be

doing a latent profile/transition analyses of parent- and child-focused ER, but since that has not been introduced yet, the term "parental profiles" could be confusing to readers.

On p.3, authors state that "Parental self- and child-focused ER are two mechanisms influencing parental well-being, children's emotional development, and children's adjustment." I caution authors against this wording which implies causality and a specific direction of effect. Parental well-being, children's emotional development, and children's adjustment may also influence parental self- and child-focused ER. On the topic of causality and directionality, I wonder if, instead of referring to parents' education level and gender as antecedents and parental exhaustion, children's internalizing problems, children's achievement, and parent–child relationship closeness as outcomes, authors should refer to these other variables as correlates of profile membership? These terms appear throughout the manuscript.

On p.3, Morris et al. (2017), which authors already cite as reference 13, would help support their point that early to mid-adolescence is a developmental period during which parental emotion support has been understudied.

Can authors please provide a citation for the sentence, "Academic demands at this stage are often emotionally charged and require considerable regulatory involvement from parents, but such involvement may change over the course of the school year" on p.3?

On p.4, authors cite Mikolajczak et al. (2023) as evidence that "The close relationship between self- and child-focused ER is observable in the short-term, as parents experience lower distress when their child becomes better regulated"—I don't think that the cited chapter presents empirical data on this.

On p.5, authors state that both reappraisal and rumination are widely studied for child-focused ER. I agree that they are widely studied for self-focused ER, but I don't believe they are widely studied for child-focused ER—that is an innovation of the present study.

On p.6, authors cite Teuber et al. (2025), who used the same data, as a study that found parents' well-being influenced how they attempt to modulate their children's emotions. Did this study's methods align with the use of this causal language?

Can authors expand on p.6 about how they think parental self- or child-focused rumination could be helpful in everyday parenting life?

The latter half of the paragraph spanning page 8-9 is a bit confusing—can authors explain what they mean that "child-focused emotional competencies, including child-focused ER, have been identified as a vulnerability factors for some parent populations and not associated for others", and that "Regulating their children can be very effortful and exhausting for parents with their self-focused ER potentially being central for their well-being, although this effect might not be the same for children's well-being."

For authors point on p.9 that "More concretely, parents' stress responses and self-focused ER are robustly tied to how children regulate their own emotions, which in turn is closely linked to their internalizing problems" I recommend adding citations. One helpful review might be Bariola et al. (2011).

Bariola, E., Gullone, E., & Hughes, E. K. (2011). Child and adolescent emotion regulation: The role of parental emotion regulation and expression. Clinical Child and Family Psychology Review, 14(2), 198–212. https://doi.org/10.1007/s10567-011-0092-5

As mentioned above, the systematic review Edler & Valentino (2024) could also be helpful for supporting the point on p.9 that parental self-focused ER is linked to emotion-related parenting behaviors specifically.

Is there any empirical evidence that authors could cite for their point on p.10 that "Interestingly, closer parent–child relationships often involve more frequent interactions, which also include child-focused rumination"?

Method

On p.11, it would be helpful for authors to cite the prior work they refer to on ER profiles in parenting. Same with authors' statement that there is moderate stability in self-focused ER and parenting behaviors.

Did authors have hypotheses about the direction of association between profiles and parental education/gender?

Could authors report more detail about the sample demographics, if possible? 62.91% of parents had a university level education, what about the rest of the sample? Was information gathered on family income and family structure (e.g., single parent status)?

What was the attrition from T1 to T2 and T3?

Why did authors only use 4/6 items from the ERQ cognitive reappraisal subscale?

What was the skew for the academic achievement variable, and for the other variables?

Can authors provide a citation for their point on p.15 that internalizing problems are a proximal outcome of children's self-focused ER? Perhaps Compas et al. (2017), referenced above.

Why did authors run a two-factor CFA for child internalizing symptoms?

Were any residuals between different items allowed to correlate in any of the authors' CFAs or the longitudinal measurement invariance models?

Authors write on p.16 that the configural, metric, and scalar longitudinal measurement invariance models were compared pairwise. Did authors compare configural to metric, and metric to scalar, using likelihood ratio tests?

On p.16, authors write "Higher entropy values (0-1)…" and I understand that they mean the range of entropy can be from 0-1, but readers may not know this and think that a 0 or a 1 (vs. a hypothetical negative value) indicates poor class separation.

Authors mention using auxiliary three-step analyses. Is this similar to the Bolck-Croon-Hagenaars method? Or more similar to Vermunt's three-step approach? If authors could elaborate on this analytic choice it would be helpful.

Results

It is very interesting that the "reappraisers" were also elevated in child-focused rumination. I wonder if authors could relabel this profile to capture that important nuance?

Similarly, looking at Figure 2, the self-ruminators were actually somewhat low in self-rumination. Could another important dimension of this grouping be parents' disengagement with self-focused reappraisal and child-focused ER?

Did authors conduct formal pairwise comparisons to conclude that the average regulators had lower self- and child-focused reappraisal compared with the those in the reappraisers profile (p.19), or were these just visual comparisons?

Discussion

It's interesting that self-ruminators had the highest stability. At the same time, it looks like a decent number of parents transitioned into the self-rumination profile. In a sense, could this indicate there was some instability in level of self-rumination?

Authors say on p.21 that "Contrary to our hypotheses, child-focused rumination aligned with child-focused reappraisal but at lower levels"—I thought the reappraisers profile was characterized by both relatively higher levels of reappraisal and child-focused rumination?

It would be helpful for authors to expand on their point that "These differences are also reflected by the respective measurement scales where self-focused rumination typically includes reflection and brooding subscales, whereas child-focused rumination is assessed through reflection only." Readers less familiar with the rumination literature could benefit from more discussion about the distinction between reflection and brooding.

I wonder if it would be helpful for authors to list all five of the child-focused rumination items to better interpret the findings.

P.24 of the discussion is another spot where authors use causal language and strongly imply that the profiles are leading to the "outcomes." I think it would be worthwhile for authors to explore the possibility that the outcomes may be contributing to the profiles. E.g., if a child has more internalizing symptoms, or a parent is experiencing more burnout, maybe that contributes to higher self-focused rumination? This is an important direction to consider, especially as it sounds like internalizing symptoms at T1 were examined in association with transitions in profiles from T1 to T2.

On p.24 authors cite Zimmer-Gembeck et al. (2022) and say that "Previous research has also shown that parental self-focused ER is not necessarily directly associated with parenting behavior but is related to children's socioemotional adjustment." This is not what the meta-analysis found; parental ER was associated directly with parenting behavior. See also Crandall et al. (2015), Edler & Valentino (2024), Zitzmann et al. (2024).

On p.25, adding the world parental before "child-focused ER" would be helpful for this sentence: "Also, children's self-focused ER can influence academic achievement, but evidence of child-focused ER remains limited."

Do authors expect that findings would have been similar if they had used a non-academic related stem for the parental child-focused ER efforts?

I think there is a typo on p.26, when authors say "Future research should include a broader range of studies, incorporating strategies such as problem-solving and avoidance," do they mean strategies instead of studies?

Hajal & Paley (2020) is a relevant study for the authors' point on p.28 about intervening via parental emotion regulation to support child development.

* TRANSPARENT PEER REVIEW: Communications Psychology uses a transparent peer review system. This means that we publish the editorial decision letters including Reviewers' comments to the authors and the author rebuttal letters online as a supplementary peer review file. However, on author request, confidential information and data can be removed from the published reviewer reports and rebuttal letters prior to publication. If your manuscript has been previously reviewed at another journal, those Reviewers' comments would not form part of the published peer review file.

** Visit Nature Research's author and referees' website at www.nature.com/authors for information about policies, services and author benefits**

Communications Psychology is committed to improving transparency in authorship. As part of our efforts in this direction, we are now requesting that all authors identified as 'corresponding author' create and link their Open Researcher and Contributor Identifier (ORCID) with their account on the Manuscript Tracking System prior to acceptance. ORCID helps the scientific community achieve unambiguous attribution of all scholarly contributions. You can create and link your ORCID from the home page of the Manuscript Tracking System by clicking on 'Modify my Springer Nature account' and following the instructions in the link below. Please also inform all co-authors that they can add their ORCIDs to their accounts and that they must do so prior to acceptance.
https://www.springernature.com/gp/researchers/orcid/orcid-for-nature-research

For more information please visit http://www.springernature.com/orcid

If you experience problems in linking your ORCID, please contact the Platform Support Helpdesk.

Version 1:

Decision Letter:

** Please ensure you delete the link to your author homepage in this e-mail if you wish to forward it to your coauthors **

Dear Ms Jordan,

Your manuscript titled "Longitudinal Profiles of Parental Self- and Child-Focused Emotion Regulation" has now been seen by our reviewers, whose comments appear below. In light of their advice I am delighted to say that we are happy, in principle, to publish a suitably revised version in Communications Psychology.

We therefore invite you to revise your paper one last time to address the remaining concerns of our reviewers and a list of editorial requests. At the same time we ask that you edit your manuscript to comply with our format requirements and to maximise the accessibility and therefore the impact of your work.

EDITORIAL REQUESTS:

Please review our specific editorial comments and requests regarding your manuscript in the attached "Editorial Requests Table". Please outline your response to each request in the right hand column. Please upload the completed table with your manuscript files as a Related Manuscript file.

If you have any questions or concerns about any of our requests, please do not hesitate to contact me.

SUBMISSION INFORMATION:

In order to accept your paper, we require the files listed here https://www.nature.com/documents/commsj-file-checklist.pdf .

OPEN ACCESS:

Communications Psychology is a fully open access journal. Articles are made freely accessible on publication. For further information about article processing charges, open access funding, and advice and support from Nature Research, please visit https://www.nature.com/commspsychol/open-access

At acceptance, you will be provided with instructions for completing the open access licence agreement on behalf of all authors. This grants us the necessary permissions to publish your paper. Additionally, you will be asked to declare that all required third party permissions have been obtained, and to provide billing information in order to pay the article-processing charge (APC).

* TRANSPARENT PEER REVIEW: Communications Psychology uses a transparent peer review system. On author request, confidential information and data can be removed from the published reviewer reports and rebuttal letters prior to

publication. If you are concerned about the release of confidential data, please let us know specifically what information you would like to have removed. Please note that we cannot incorporate redactions for any other reasons.

* CODE AVAILABILITY: All Communications Psychology manuscripts must include a section titled "Code Availability" at the end of the methods section. We require that the custom analysis code supporting your conclusions is made available in a publicly accessible repository at this stage; please choose a repository that generates a digital object identifier (DOI) for the code; the link to the repository and the DOI must be included in the Code Availability statement. Publication as Supplementary Information will not suffice.

* DATA AVAILABILITY:
All Communications Psychology manuscripts must include a section titled "Data Availability" at the end of the Methods section. More information on this policy, is available in the Editorial Requests Table and at http://www.nature.com/authors/policies/data/data-availability-statements-data-citations.pdf.

Please use the following link to submit the above items:
Link Redacted
** This url links to your confidential home page and associated information about manuscripts you may have submitted or be reviewing for us. If you wish to forward this email to co-authors, please delete the link to your homepage first **

We hope to hear from you within two weeks; please let us know if you need more time.

Best regards,

Jennifer Bellingtier

Jennifer Bellingtier, PhD
Senior Editor
Communications Psychology

Nida Ali, PhD
Editorial Board Member
Communications Psychology
orcid.org/0000-0003-4921-7940

REVIEWER EXPERTISE:
longitudinal research, latent profile analyses and latent transition analyses, parent- child relationships

REVIEWERS' COMMENTS:

Reviewer #1 (Remarks to the Author):

Thank you for addressing all of my comments, including additional analyses and clarifications in the manuscript. The authors did a great job and the article will hopefully make a meaningful contribution to the literature.

Signed
Aljoscha Dreisoerner

Reviewer #2 (Remarks to the Author):

“Longitudinal Profiles of Parental Self- and Child-Focused Emotion Regulation” is a strong and improved manuscript. It is clear that the authors thoughtfully and carefully attended to their revisions. I have no further suggestions for the authors.

** Visit Nature Research's author and referees' website at www.nature.com/authors for information about policies, services and author benefits**

Open Access This Peer Review File is licensed under a Creative Commons Attribution 4.0 International License, which permits use, sharing, adaptation, distribution and reproduction in any medium or format, as long as you give appropriate credit to the original author(s) and the source, provide a link to the Creative Commons license, and indicate if changes were made.
In cases where reviewers are anonymous, credit should be given to 'Anonymous Referee' and the source.
The images or other third party material in this Peer Review File are included in the article's Creative Commons license, unless indicated otherwise in a credit line to the material. If material is not included in the article's Creative Commons license and your intended use is not permitted by statutory regulation or exceeds the permitted use, you will need to obtain permission directly from the copyright holder.
To view a copy of this license, visit https://creativecommons.org/licenses/by/4.0/

Comments Reviewer #1

1. *The authors present a latent class profile analysis from two timepoints using a dataset on emotional regulation responses (reappraisal and rumination) and their association with parental exhaustion, children's Internalizing Problems, children's achievement. The article is well written and the topic deserves attention. Other strengths include the second data point and sample size. I think the introduction could benefit from more structure. The latent class profile analysis might reflect the Salsa effect which would point to the lack of different profiles in the population. I have attached my comments below and hope the authors find them helpful. Please note that my perception of the manuscript is subjective and my feedback includes only suggestions. I wish the authors the best of luck and success with their research.*

   Authors' response: Thank you very much for taking the time to carefully review our manuscript. We greatly appreciate your positive evaluation of our manuscript as well as your constructive feedback for further revision. Your methodological and argumentative comments were very helpful, and we have addressed them step by step below (changes, including restructured paragraphs, are marked in orange; newly added sections are additionally highlighted in yellow). We have also thoroughly tested a potential Salsa effect, which could not be identified in our data. Instead, we could clearly show three distinct profiles (i.e., differing in their quality). We hope we have addressed your suggestions and concerns appropriately.

2. *Overall, I found the introduction far too long and as a result a bit disjointed and unfocused. You introduce a lot of concepts and discuss their antecedents and outcomes, but in my reading, the main contribution of the current study becomes harder to identify that way.*

   Authors' response: Thank you very much for your helpful comment. We have substantially shortened and restructured the entire theory section. In doing so, we aimed to better highlight the manuscript's main focus, as you suggested, by elaborating the relationship between parental self- and child-focused emotion regulation and the relevant literature. In addition, we reduced the sections on correlates, which we included as antecedents and outcomes in our analyses, to two paragraphs. We have included these paragraphs below for your review. We hope the theory section now reads more smoothly and is overall more concise and focused.

   Changes in manuscript: "Profiles of parent self- and child-focused emotion regulation may be associated with sociodemographic factors such as parental gender and educational level. Stereotypically, mothers' parenting practices are more strongly associated with emotionality and nurturance in Western cultures[54], although evidence of parents' emotion regulation is mixed, with some studies indicating no differences in parents' emotional socialization (for a broad overview of recent work, see[55]), and others suggesting higher involvement of mothers including more supportive strategies[56–58]. Self-focused rumination is also consistently higher among females than males[4,49], whereas in self- and child-focused reappraisal, gender differences are not reported as often[3,59–61]. Another sociodemographic variable that potentially predicts parental

emotion regulation profiles is parents' educational level, an indicator of socioeconomic status (SES), which is robustly associated with individuals' health[62,63]. Parents likely also experience more stress and reduced well-being when experiencing financial hardship[41], which may in turn be related to parents' strategies for self- and child-focused emotion regulation.

Patterns of parents' self- and child-focused emotion regulation are likely interconnected with family well-being and relationship closeness, with prior work predominantly focusing on links to child development[8,52,64]. Parents' self-focused emotion regulation and parenting practices are robustly tied to how children regulate their own emotions, which in turn is closely linked to their internalizing problems, characterized by inwardly oriented affective disruptions[7,41,65,66]. Overall, parental self- and child-focused emotion regulation has been repeatedly associated with child adjustment[8], which presumably encompasses academic achievement during adolescence[32,47,67]. Less attention has been given to parental correlates including parental exhaustion (i.e. persistent parenting-related stress and an early stage of parental burnout[68,69]), but evidence links parental exhaustion to lower self-focused reappraisal (for a meta-analysis, see [56]), higher self-focused rumination[71], and disruptions in child development[23,72,73]. Lastly, parent–child relationship closeness may be associated with parental self-and child-focused emotion regulation, as higher emotion regulation skills are related to more secure attachment[74,75], and authoritative parenting characterized by warmth and structure[28,76], whereas self-focused emotional instability has been linked with parental distancing and fewer or lower quality parent–child interactions[21,77]."

3. *In addition, I am not sure the introduction reflects a full account of the studies describing co-regulation strategy use of parents and their children, which I understand is the main focus of the current study. What does the current literature say on how parents regulate themselves and their children's emotions at the same time?*

   Authors' response: Thank you for this important comment. There is limited evidence on parental self- and child-focused emotion regulation. However, child-focused emotion regulation closely overlaps with the concepts of co-regulation, emotion socialization and emotion-related parenting behaviors. Therefore, we included a section reviewing the literature on parents' self-focused emotion regulation and emotion-related parenting. To clarify that the main focus of the study is the association between self- and child-focused emotion regulation, we moved this section to the beginning of the manuscript. In addition, we include a sentence acknowledging the connection to these overlapping concepts.

   Changes in manuscript: "Consider, for instance, an adolescent who comes home upset after receiving a poor report card and is facing the possibility of repeating the year. The parent may attempt to regulate their own negative emotions (e.g., disappointment or frustration) while also helping the child downregulate their emotional distress. These mechanisms are classified under the concept of emotion regulation, which refers to the process by which individuals modify emotional states in pursuit of specific goals[10,11]. Such regulatory goals can be primarily directed

toward one's own emotions (self-focused emotion regulation) or another person's emotions (other-focused emotion regulation)[12], with these goal directions being activated separately or coactivated. The literature on parental self- and child-focused emotion regulation is closely intertwined with overlapping concepts such as coregulation, emotion socialization, and interpersonal emotion regulation[13], where prior research indicates interrelations between how parents regulate their own and their children's emotions[14,15]. More precisely, parents with higher self-focused emotion regulation skills or capacities are more likely to respond supportively to children's emotions, be more involved and sensitive in their parenting, and use fewer negative and unsupportive practices, including hostility and harshness[8,16–18]. Conversely, parents who engage more in self-focused emotion dysregulation tend to use more non-supportive and disengaged regulation practices and are more likely to engage in child maltreatment[8,17,19–22]."

4. *The writing in the section "Self- and Child-Focused Emotion Regulation" is a bit disjointed (particularly in the first paragraph)*

Authors' response: Thank you for pointing this out. We restructured the paragraphs and, in line with your previous comments, added a more in-depth discussion of current theorizing and available evidence on how parents regulate their own and their children's emotions. We hope the reading flow has improved and that the section now provides a clearer overview of the existing literature.

Changes in manuscript: "Consider, for instance, an adolescent who comes home upset after receiving a poor report card and is facing the possibility of repeating the year. The parent may attempt to regulate their own negative emotions (e.g., disappointment or frustration) while also helping the child downregulate their emotional distress. These mechanisms are classified under the concept of emotion regulation, which refers to the process by which individuals modify emotional states in pursuit of specific goals[10,11]. Such regulatory goals can be primarily directed toward one's own emotions (self-focused emotion regulation) or another person's emotions (other-focused emotion regulation)[12], with these goal directions being activated separately or coactivated. The literature on parental self- and child-focused emotion regulation is closely intertwined with overlapping concepts such as coregulation, emotion socialization and interpersonal emotion regulation[13], where prior research indicates interrelations between how parents regulate their own and their children's emotions[14,15]. More precisely, parents with higher self-focused emotion regulation skills or capacities are more likely to respond supportively to children's emotions, be more involved and sensitive in their parenting, and use fewer negative and unsupportive practices, including hostility and harshness[8,16–18]. Conversely, parents who engage more in self-focused emotion dysregulation tend to use more non-supportive and disengaged regulation practices and are more likely to engage in child maltreatment[8,17,19–22].

Parents' self- and child-focused emotion regulation facilitate children's emotion regulation development and adjustment[9,24,25], as reflected in the Tripartite Model through the mechanisms of *observation* and *parenting practices*[7]. Observation refers to the process by which children learn from observing and imitating their parents' emotional behaviors (e.g., parents' self-focused emotion regulation), whereas parenting practices describe parents' direct responses to their children's emotions, including child-focused emotion regulation. Children's need for parental support varies across development, and as they enter adolescence, they increasingly seek greater autonomy, influence how parents regulate their emotions, and draw on self-regulation[26,27]. Nonetheless, parents remain an important source of emotional support, and their nurturance is associated with higher well-being in their adolescent child[9,28]. During early and mid-adolescence, parental regulatory efforts adapt to children's changing needs, and parenting tasks shift toward fostering adolescents' independence[27,28]. Consequently, direct support (e.g., child-focused emotion regulation) likely becomes more tailored to critical developmental tasks, such as helping adolescents manage academic and performance-related pressures[29]. At the same time, parents' self-focused emotion regulation remains an important source of social learning through modeling[7,30]. Reflecting this shift, we focused on child-focused emotion regulation during children's negative achievement emotions, given the high prevalence of negative emotions in emotion regulation goals and the importance of achievement contexts in adolescence[29,31]. We focused on parents of children in Grades 6–9 because (a) early to mid-adolescence is marked by biological changes that make this stage a particularly emotionally vulnerable one requiring parental support[28,32,33], and (b) after the transition to secondary education, academic demands and maladaptive patterns of emotional development increase, on average[34,35]."

5. *I am not sure I would call rumination a strategy without first introducing the difference between adaptive and maladaptive coping. Rumination is a symptom in many disorders and referring to it as a strategy without this qualification reads like people choose to ruminate, which they most likely do subconsciously.*

   Authors' response: Thank you very much for this comment. In response, we now introduce rumination at the beginning of the introduction and note that it is often considered a symptom or correlate of psychopathology. We also restructured the paragraph on reappraisal and rumination to clarify how these processes may be used as emotion-regulation strategies, while also highlighting their links to psychological disorders and their potentially nonconscious nature. However, we refrained from using the terms "adaptive and maladaptive coping" because introducing coping would require additional definitions and conceptual distinctions, and in the revised manuscript we aimed to keep terminology to a minimum. Still, we agree that the coping literature overlaps with the emotion regulation literature, and we distinguish the two in line with Gross (2024), with both falling under affect regulation, but coping typically referring to regulation over longer time periods than emotion regulation. We hope these revisions fully address your comment.

Gross, J. J. (2024). Conceptual foundations of emotion regulation. In J. J. Gross & B. Q. Ford (Eds.), *Handbook of emotion regulation* (3rd ed., pp. 3–12). Guilford Press.

Changes in manuscript: "Considering the earlier example of a child being upset about a poor report card, a parent might regulate their own emotions by reminding themselves that this situation does not define their child's future (self-focused reappraisal) or by dwelling on thoughts such as "I must be doing something wrong as a parent" (self-focused rumination). In the child-focused context, the parent could help the child reinterpret the setback as an opportunity to learn (child-focused reappraisal) or direct the child's attention toward the failure by repeatedly questioning what went wrong (child-focused rumination). Reappraisal and rumination regulate early stages in the emotion-generation process before response modulations, with reappraisal being categorized under processes of cognitive change and rumination under attentional deployment in the extended process model of emotion regulation[1,2,11]. Habitually, higher levels of self-focused reappraisal are often associated with higher well-being, stress recovery, and psychological adjustment (for a review, see [26]), whereas higher levels of self-focused rumination are associated with increased negative mood and psychopathology, including symptoms of depression and anxiety[4,6].

In everyday parenting life, however, reappraisal and rumination can sometimes deviate from their habitual associations and may be helpful in some situations and unhelpful in others[12]. Despite the consistent benefits of self-focused reappraisal, it can, for example, be less effective in situations of high-emotionality or when cognitive resources are limited[37–39]. Self-focused rumination, on the other hand, has been described by individuals as a strategy to process and reflect on negative emotions to feel more in control of a situation[4,40]. Nevertheless, given its close link to disorders such as depression, the intense self-reflection involved in self-focused rumination can reflect individual's avoidance of situations or responsibilities, and it often occurs unconsciously or involuntarily[4,6,40]. However, most research on reappraisal and rumination has been conducted in self-focused contexts, and the extent to which results can be applied to interpersonal contexts, especially parenting, is unclear. Longitudinal profiles of parental reappraisal and rumination can tackle questions on their coexistence, address how such coexistence in turn is related to family correlates, and reveal habitual differences in the use of self- and child-focused strategies."

6. *One key Table isn't included: What are the descriptive statistics of important variables (outcomes, antecedents) by latent profile class. See* https://doi.org/10.1038/s41398-021-01508-2 *Table 3 for an example (please also include rumination and reappraisal)*

Authors' response: Thank you for this suggestion and for providing the example table. In response, we added a table reporting the means for all antecedents, outcomes, and profile-defining variables, along with ANOVAs or chi-square tests examining whether the profiles differ significantly. We reported these results for most variables at Time 1, and we included a second

set of results at Time 2 for the core variables assessed at both time points. This table has been added to the manuscript as Table 2. We hope it addresses your suggestion and includes all relevant constructs.

Changes in manuscript: "In Table 2, we report profile demographics for the emotion regulation strategies included in the profiles and the profile correlates. Differences between profiles were tested using analyses of variance (ANOVAs) for continuous variables and chi-square tests for categorical variables."

7. *Please carefully check if the solution does not reflect the Salsa effect, which just forces a single population into separate latent classes on one outcome variable. I could not verify this from the existing tables https://pmc.ncbi.nlm.nih.gov/articles/PMC7746621/.*

   I now see Figure 1 and I do have the suspicion that your solution represents the Salsa effect that divided groups into high reappraisal, moderate reappraisal and low reappraisal. Since reappraisal and rumination are strongly negatively correlated too you might have gotten the current outcome. Please discuss. If the Salsa effect is present, a variable oriented approach like multi-level modeling might be more suited to the current data or could at least complement the current analysis as a robustness check.

   Authors' response: Thank you for raising this concern and for your constructive suggestions on how to proceed should a Salsa effect be present in our data. Prior to finalizing the first manuscript draft, we explicitly evaluated whether our solution showed evidence of a Salsa effect and found no indication of this pattern in our data. To make profile distinctiveness easier to assess visually, we also revised Figure 1 to a line graph (see also below). In deciding between the three- and four-profile solutions (e.g., elbow plots suggested bends at three and four profiles), profile distinctiveness was one of the important criteria. As we state in the manuscript on p. 16, the fourth profile did not add a qualitatively distinct pattern but instead appeared to be a quantitative variant of Profile 1 in several indicators, which was one criterium that supported retaining the three-profile solution. In accordance with Marsh et al. (2009), we identified successful classification into groups with qualitatively and quantitatively different profiles, which we found in our three-profile solution.

   To carefully address your concerns during the revision, we re-checked the distributions of the indicator variables and found no issues that would affect the Salsa effect. We also re-estimated the models using increased multiple random starts (e.g., STARTS = 1,600 400), consistent with the recommendation to use at least twice as many starts to confirm the solution. The output indicated replication of the best loglikelihood for both waves, and the results did not change across waves, except that the ordering of the profiles switched. Profile sizes and profile characteristics were identical. These additional analyses therefore support the robustness of the profile solution. We have updated the corresponding output files in our OSF repository.

Regarding your comment on the negative association between reappraisal and rumination, we want to note that these constructs are conceptually distinct and therefore not expected to map onto a single factor. This is also reflected in our data, where the manifested association between self-focused reappraisal and self-focused rumination is small to medium ($r = -.19^{***}$), and child-focused reappraisal even correlates positively with child-focused rumination ($r = .24^{***}$). To fully address this concern, we investigated it by inverting rumination in a test figure (i.e., multiplied scores by −1), and the profile shapes still did not align consistent with a Salsa effect, instead showing for example a clear crossing pattern due to the positive association between child-focused rumination and child-focused reappraisal. Additionally, we ran a one-factor CFA of the emotion regulation strategies and statistically compared it with our four-factor CFA. The one-factor CFA showed poor model fits and a significantly worse fit than the four-factor model with $\Delta\chi^2(6) = 3324$, $p < .001$. Taken together, none of our analyses indicated the presence of the Salsa effect in our data. However, we greatly appreciate your remarks and your careful attention to the statistical analyses, and hope our additional analyses and clarifications address your concerns thoroughly enough.

Marsh, H. W., Lüdtke, O., Trautwein, U., & Morin, A. J. S. (2009). Classical latent profile analysis of academic self-concept dimensions: Synergy of person- and variable-centered approaches to theoretical models of self-concept. *Structural Equation Modeling: A Multidisciplinary Journal, 16*(2), 191–225. https://doi.org/10.1080/10705510902751010

Changes in manuscript: "We carefully assessed profile distinctiveness through visual inspection and by re-estimating the solution with multiple random starts[100], and found no indication of the presence of a Salsa effect in our data (i.e., classes reflect mainly quantitative differences in the indicators)."

Figure 1

A. Profiles of Parental Self- and Child-Focused Emotion Regulation Strategies at T_1

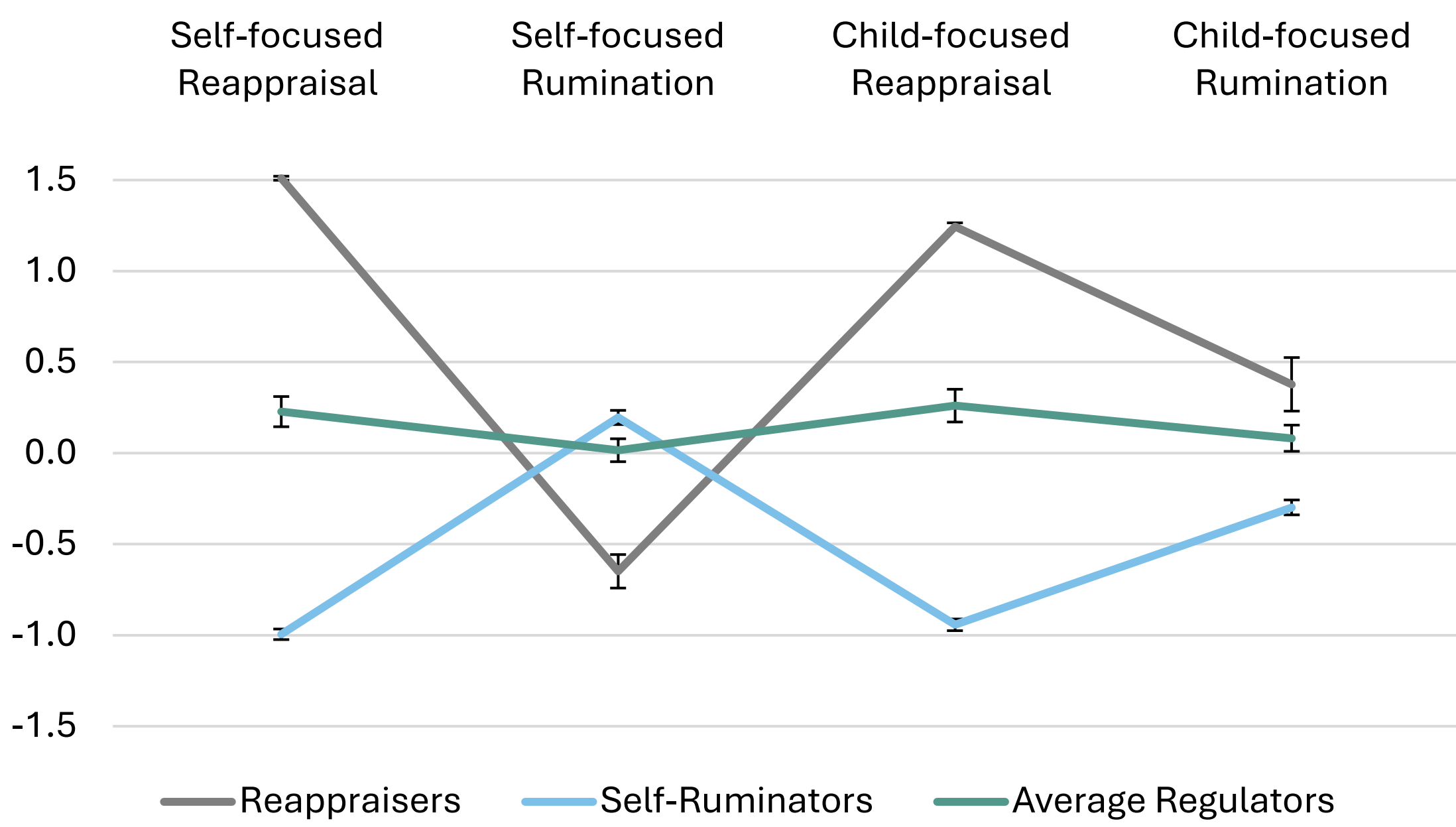

B. Profiles of Parental Self- and Child-Focused Emotion Regulation Strategies at T_2

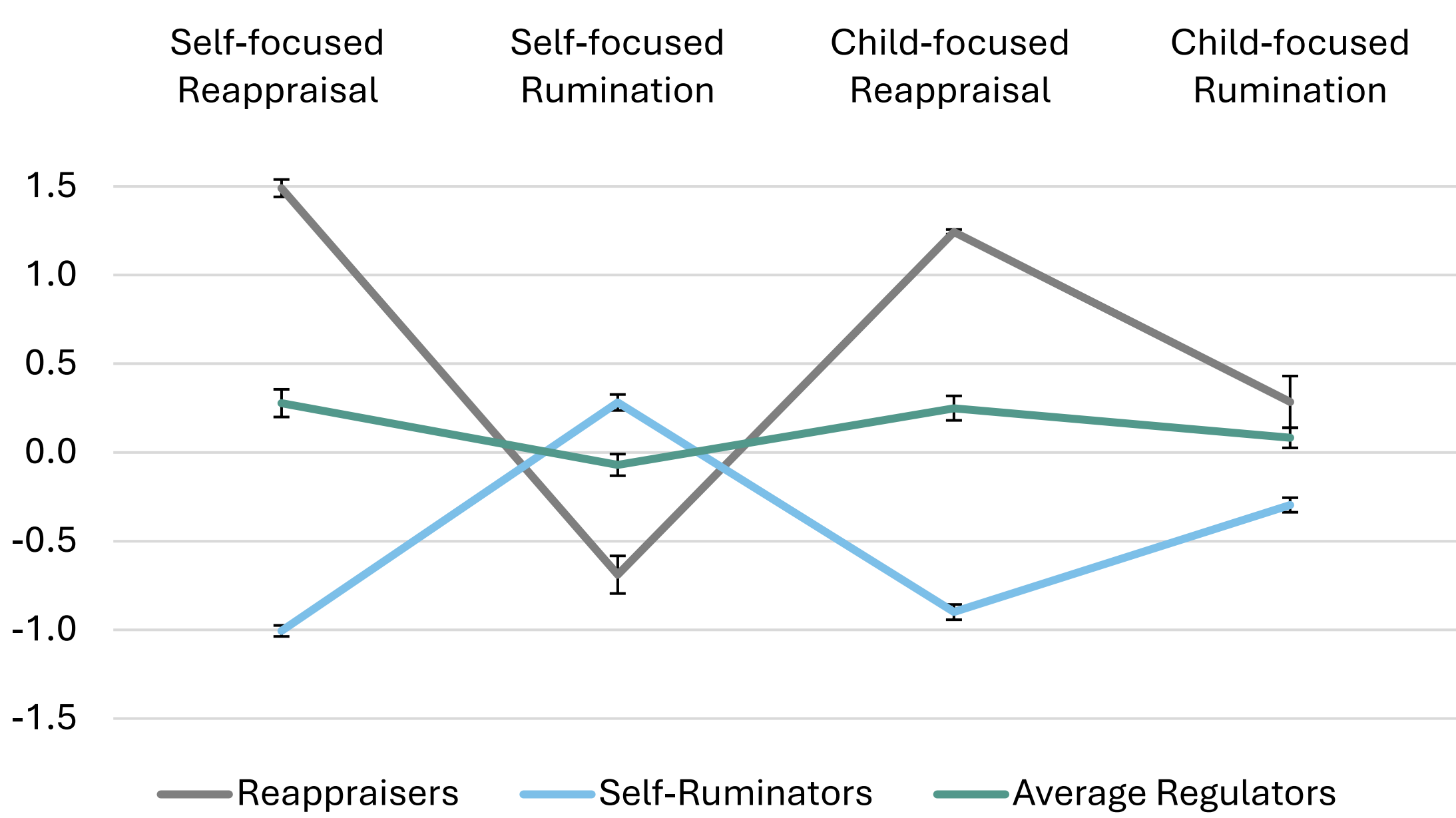

Note. T_1 = time point one; T_2 = time point two; each emotion regulation strategy in the profiles is reported with error bars representing ±1 standard error of the mean, *N* = 1,046 participants.

8. *The labels for the bars in Figure 1, make the graph hard to understand*

   Authors' response: Thank you for pointing this out. We have revised Figure 1 to a line graph to make it easier for readers to examine effects such as the Salsa Effect and in accordance with the guidelines of *Communications Psychology*. We hope the labels are better to comprehend in that way as well.

9. *Tables dont explain abbreviations currently*

   Authors' response: Thank you for your comment. We carefully checked the tables again to ensure that all abbreviations are explained in the table notes.

10. *I would like to see a table that lists key variables by latent profile class (also demographics)*

    Authors' response: Thank you for the comment. We think this comment is in accordance with a similar one from above, where we computed ANOVA's and chi-square tests to statistically test differences of key variables between profiles. We included all results in Table 2.

    Changes in manuscript: "In Table 2, we report profile demographics for the emotion regulation strategies included in the profiles and the profile correlates. Differences between profiles were tested using analyses of variance (ANOVAs) for continuous variables and chi-square tests for categorical variables."

11. *Maybe I missed it, but what was the dropout in this study?*

    Authors' response: Thank you for this question. We have now included a more detailed description of attrition across waves and the composition of the datasets. Because only two outcomes from Time point 3 were used in the present study, the primary dataset consisted of data from Time point 1 and Time point 2. We have added a more detailed overview of the procedure, including dropouts and missing data for the different datasets used in the analyses.

    Changes in manuscript: "We collected longitudinal data at three time points (T_1 in September 2023, T_2 in January 2024, T_3 in May 2024), following parents across one school year. After cleaning the data and excluding nonmatchable records, 1,359 participants completed the survey at T_1, and 1,046 participants provided data at T_1 and T_2, indicating an attrition rate of 23.03% (n = 313). Thus, the final sample for the main analyses (T_1–T_2 dataset) consisted of N = 1,046 parents of sixth–ninth graders residing in the US. At the third time point, 875 participants could be matched to the T_1–T_2 dataset. Missing data in the T_1–T_2 dataset were 0.60%, and missing data in the dataset including all three time points were 4.62%."

12. *In latent class analysis, it can become an issue when one class is particularly small. Please discuss the methodological and theoretical implications of this some more.*

    Authors' response: We appreciate this comment and have expanded our discussion of small profiles and also justified why we consider the *reappraiser* profile to be qualitatively meaningful.

    Changes in manuscript: "The three profiles differed substantially in size with the *average regulator* profile being the largest profile (n_{T1} = 692; n_{T2} = 643), and the *reappraiser* profile being the smallest (n_{T1} = 91; n_{T2} = 78). When interpreting small groups, such as the *reappraiser* profile, it is important to consider their qualitative contribution, as estimates for small profiles can be less precise and statistical power can be reduced[102]. However, the *reappraisers* profile provides valuable insight into a potentially highly functional emotion regulation pattern and does not fall below common minimum-size guidelines (i.e., < 5% of the sample or < 50 cases) [103]. The *self-ruminators* profile represented the second largest group and was the only one to increase in size over time (n_{T1} = 264; n_{T2} = 326). These results may reflect rising stress and regulatory demands as the school year progresses, given that T_1 occurred at the start of children's school year. Interestingly, the overall sample mean of self-focused rumination was very stable across both time points, whereas the *self-ruminators* showed a slight increase in self-focused rumination, demonstrating a central advantage of person-centered approaches in identifying subgroup-specific changes that are not captured by aggregate means. The unequal profile sizes might partly also reflect the limited range of the emotion regulation strategies assessed, as different profiles may have emerged if additional strategies had been included. Still, our findings underscore the need for tailored interventions that specifically target rumination to counteract its negative effects."

13. *The limitations currently don't address the inherent limitations of latent class analysis*

    Authors' response: Thank you for your comment. We included a statement on the inherent limitations of latent class analyses in the beginning of the limitations section.

    Changes in manuscript: "Although the person-centered approach is well-suited for identifying subgroups, LPA and LTA assume that the observed distribution in the sample reflects the distribution of underlying populations, reduce variances as groups are estimated to maximize distinctiveness, and the methodological process includes subjective decisions regarding the final models[100,103,110]."

14. *For readability, I would recommend not abbreviating emotional regulation*

    Authors' response: Thank you very much for this remark. Indeed, abbreviating the term emotion regulation may be less clear to readers who are not familiar with this specific literature. For this reason, we followed your advice and removed the abbreviation throughout the manuscript.

Comments Reviewer #2

1. *Thank you for the opportunity to review this manuscript, which presents interesting findings that contribute to the literature on intra and interpersonal emotion regulation. I think that the method and results sections are overall well-written and clear, but the introduction and discussion sections have room for improvement. A number of my comments revolve around (1) authors implying that there is no or limited literature on topics that are well-researched and (2) authors' use of causal language. I hope that authors find these revisions helpful as they move forward this manuscript.*

   Authors' response: Thank you very much for your positive review of our manuscript and for taking the time to look at our article in detail. Your thoughtful recommendations were very helpful in enhancing the article, and we especially appreciated your advice on integrating the broader parenting literature, which we believe significantly improved the quality of our work. Below, we address your comments in more depth, point by point (changes, including restructured paragraphs, are marked in orange; newly added sections are additionally highlighted in yellow). We hope our revisions sufficiently address your suggestions.

2. *Authors write that patterns of relationships between the variables they focus on remain "unexplored"—a term such as "underexplored" would be better, though that also is not quite accurate because there are large literatures on relations between the variables studied in this paper. See below for a few examples that authors do not cite in the paper (critical reviews that authors do helpfully cite are Zimmer-Gembeck et al., 2022; Zitzmann et al., 2024; and Brandao et al., 2024). I don't think that these need to be cited in the abstract specifically, but citing somewhere the four reviews/meta-analyses below on parenting ER frameworks would help support the current study.*

   Crandall, A., Deater-Deckard, K., & Riley, A. W. (2015). Maternal emotion and cognitive control capacities and parenting: A conceptual framework. Developmental Review, 36, 105–126. https://doi.org/10.1016/j.dr.2015.01.004

   Hajal, N. J., & Paley, B. (2020). Parental emotion and emotion regulation: A critical target of study for research and intervention to promote child emotion socialization. Developmental Psychology, 56(3), 403–417. https://doi.org/10.1037/dev0000864

   Zhang, X., Beatty, A., Abela, K., Fernandes Melo, M., Kenny, M., Atkinson, L., & Gonzalez, A. (2023). Assessing parental emotion regulation in the context of parenting: A systematic review. Developmental Review, 69, 1–37. https://doi.org/10.1016/j.dr.2023.101092

   Edler, K., & Valentino, K. (2024). Parental self-regulation and engagement in emotion socialization: A systematic review. Psychological Bulletin, 150(2), 154–191. https://doi.org/10.1037/bul0000423

Compas, B. E., Jaser, S. S., Bettis, A. H., Watson, K. H., Gruhn, M. A., Dunbar, J. P., Williams, E., & Thigpen, J. C. (2017). Coping, emotion regulation, and psychopathology in childhood and adolescence: A meta-analysis and narrative review. Psychological Bulletin, 143(9), 939–991. https://doi.org/10.1037/bul0000110

Authors' response: Thank you very much for your comment and for the highly relevant literature suggestions. We have included all suggested references in the revised manuscript and have adapted the Introduction in line with your and the other reviewers' comments by incorporating and elaborating on relevant literature on parents' emotion regulation and emotion socialization practices. In addition, we clarified the wording in the Abstract and now refer to patterns of self- and child-focused reappraisal and rumination as being underexplored.

Changes in manuscript: "Parenting is an emotion-eliciting experience that requires parents to navigate the dual demands of regulating their own and their children's emotions, drawing on regulatory strategies such as reappraisal and rumination. However, patterns of self- and child-focused reappraisal and rumination and their associations with parental exhaustion, children's internalizing problems, children's academic achievement, and parent–child relationships remain underexplored."

3. *On p.3, authors write that "longitudinal patterns of parental self- and child-focused ER have not been explored." Child-focused ER, as the authors describe, is very similar to the concept of parental emotion socialization (efforts to change children's emotions via parents' responses to children's emotions). Edler & Valentino (2024) summarize literature on parental ER and parental emotion socialization behaviors, and cover several articles that present longitudinal associations between parental ER and parental supportive and nonsupportive reactions to children's emotions: for example, see Price & Kiel (2022). Although I understand that authors may mean that few (or no) studies have conducted profile analyses with the specific variables they use, and examined whether these profiles change over time, this literature is important to acknowledge—as currently written, the statement "longitudinal patterns of parental self- and child-focused ER have not been explored" is misleading.*

   Price, N. N., & Kiel, E. J. (2022). Longitudinal links among mother and child emotion regulation, maternal emotion socialization, and child anxiety. Research on Child and Adolescent Psychopathology, 50(2), 241–254. https://doi.org/10.1007/s10802-021-00804-1

   Authors' response: Thank you for pointing out this misleading phrase. Indeed, as we also corrected in the Abstract, it is the specific profiles of parental self- and child-focused reappraisal and rumination that have not been investigated before. We therefore deleted the sentence from the introduction and incorporated your proposed citations later in the theory, alongside

previously mentioned literature, to provide an overview of existing studies on parental emotion regulation and emotion-related parenting behaviors/socialization behaviors.

Changes in manuscript: "Consider, for instance, an adolescent who comes home upset after receiving a poor report card and is facing the possibility of repeating the year. The parent may attempt to regulate their own negative emotions (e.g., disappointment or frustration) while also helping the child downregulate their emotional distress. These mechanisms are classified under the concept of emotion regulation, which refers to the process by which individuals modify emotional states in pursuit of specific goals[10,11]. Such regulatory goals can be primarily directed toward one's own emotions (self-focused emotion regulation) or another person's emotions (other-focused emotion regulation)[12], with these goal directions being activated separately or coactivated. The literature on parental self- and child-focused emotion regulation is closely intertwined with overlapping concepts such as coregulation, emotion socialization and interpersonal emotion regulation[13], and prior work indicates interrelations between how parents regulate their own and their children's emotions[14,15]. More precisely, parents with higher self-focused emotion regulation skills and capacities are more likely to respond supportively to children's emotions, be more involved and sensitive in their parenting, and use fewer negative and unsupportive practices, including hostility and harshness[8,16–18]. Conversely, parents who engage more in self-focused emotion dysregulation tend to use more non-supportive and disengaged regulation practices and are more likely to engage in child maltreatment[8,17,19–22]."

4. *On p.3, authors refer to "parental profiles" without explaining what they mean by profiles. Later, they explain they will be doing a latent profile/transition analyses of parent- and child-focused ER, but since that has not been introduced yet, the term "parental profiles" could be confusing to readers.*

   Authors' response: Thank you for this remark. We have rephrased the sentences in the introduction to refer to patterns of emotion regulation strategy use targeting parents' own versus their children's emotions, so that the more technical term "profiles" does not appear before we clarify that we conduct a profile analysis.

   Changes in manuscript: "Understanding patterns of parental self- and child-focused emotion regulation has important empirical and practical implications because different combinations may help explain variability in parental and child well-being, as well as the parent–child relationship, thereby informing the development of tailored interventions."

5. *On p.3, authors state that "Parental self- and child-focused ER are two mechanisms influencing parental well-being, children's emotional development, and children's adjustment." I caution authors against this wording which implies causality and a specific direction of effect. Parental well-being, children's emotional development, and children's adjustment may also influence*

parental self- and child-focused ER. On the topic of causality and directionality, I wonder if, instead of referring to parents' education level and gender as antecedents and parental exhaustion, children's internalizing problems, children's achievement, and parent–child relationship closeness as outcomes, authors should refer to these other variables as correlates of profile membership? These terms appear throughout the manuscript.

Authors' response: Thank you for making this point. We have revised the wording accordingly and now emphasize that these mechanisms are "associated with" parental well-being, emotional development, and children's adjustment. As you suggest, throughout the Introduction and in the Discussion we describe these links as "correlates," and we have double-checked the entire manuscript to remove any causal language. However, because antecedents and outcomes are examined using different statistical approaches, we keep these technical terms where needed in the Method and Results sections to clearly distinguish the two statistical tests. In addition, we kept these wordings for the Method and Results sections because the longitudinal design includes a temporal ordering among variables, and our analyses test whether earlier measures predict later outcomes.

Changes in manuscript: "Furthermore, we examined correlates of these profiles including parents' educational level, parents' gender, parental exhaustion, children's internalizing problems, children's achievement, and parent–child relationship closeness."

6. *On p.3, Morris et al. (2017), which authors already cite as reference 13, would help support their point that early to mid-adolescence is a developmental period during which parental emotion support has been understudied.*

   Authors' response: Thank you for pointing out the reference here. We included the citation for the argument as you suggested.

7. *Can authors please provide a citation for the sentence, "Academic demands at this stage are often emotionally charged and require considerable regulatory involvement from parents, but such involvement may change over the course of the school year" on p.3?*

   Authors' response: Thank you for this comment. When shortening and relocating parts of the manuscript in accordance with other reviewer comments, we deleted this sentence to keep the Introduction more focused on the main concepts of self- and child-focused reappraisal and rumination. However, later in the manuscript we include a section on academic demands and parental involvement, where we cite relevant literature to support these points.

   Changes in manuscript: "Reflecting this shift, we focused on child-focused emotion regulation during children's negative achievement emotions, given the high prevalence of negative emotions in emotion regulation goals and the importance of achievement contexts in

adolescence[29,31]. We focused on parents of children in Grades 6–9 because (a) early to mid-adolescence is marked by biological changes that make this stage a particularly emotionally vulnerable one requiring parental support[28,32,33], and (b) after the transition to secondary education, academic demands and maladaptive patterns of emotional development increase, on average[34,35]."

8. *On p.4, authors cite Mikolajczak et al. (2023) as evidence that "The close relationship between self- and child-focused ER is observable in the short-term, as parents experience lower distress when their child becomes better regulated"—I don't think that the cited chapter presents empirical data on this.*

   Authors' response: Thank you for your comment. While restructuring the Theory section, we removed this sentence. However, we integrated the point you are referring to into the Discussion when addressing the bidirectionality between parental emotion regulation and profile outcomes. As you note, to the best of our knowledge, this argument has not yet been empirically tested using this specific terminology. Therefore, we rephrased the sentence to better convey the underlying theoretical rationale.

   Changes in manuscript: "Therefore, it is important to consider that our measured correlates may also contribute to the profiles. For example, higher child internalizing problems or greater parental exhaustion might increase parents' self-focused rumination, whereas in the *reappraisers* profile, lower parental exhaustion and fewer child internalizing problems might be linked to parents' greater capacity to use self- and child-focused reappraisal[39]. Similarly, parents have been posited to experience lower distress when their child becomes better regulated[23]."

9. *On p.5, authors state that both reappraisal and rumination are widely studied for child-focused ER. I agree that they are widely studied for self-focused ER, but I don't believe they are widely studied for child-focused ER—that is an innovation of the present study.*

   Authors' response: We appreciate this remark. In line with this, and with Reviewer 2's comments 2 and 5, we restructured the section on reappraisal and rumination. As part of this revision, we removed the phrasing you refer to. Below you find the revised paragraph.

   Changes in manuscript: "Considering the earlier example of a child being upset about a poor report card, a parent might regulate their own emotions by reminding themselves that this situation does not define their child's future (self-focused reappraisal) or by dwelling on thoughts such as "I must be doing something wrong as a parent" (self-focused rumination). In the child-focused context, the parent could help the child reinterpret the setback as an opportunity to learn (child-focused reappraisal) or direct the child's attention toward the failure by repeatedly questioning what went wrong (child-focused rumination). Reappraisal and rumination regulate early stages in the emotion-generation process before response

modulations, with reappraisal being categorized under processes of cognitive change and rumination under attentional deployment in the extended process model of emotion regulation[1,2,11]. Habitually, higher levels of self-focused reappraisal are often associated with higher well-being, stress recovery, and psychological adjustment (for a review, see [26]), whereas higher levels of self-focused rumination are associated with increased negative mood and psychopathology, including symptoms of depression and anxiety[4,6].

In everyday parenting life, however, reappraisal and rumination can sometimes deviate from their habitual associations and may be helpful in some situations and unhelpful in others[12]. Despite the consistent benefits of self-focused reappraisal, it can, for example, be less effective in situations of high-emotionality or when cognitive resources are limited[37–39]. Self-focused rumination, on the other hand, has been described by individuals as a strategy to process and reflect on negative emotions to feel more in control of a situation[4,40]. Nevertheless, given its close link to disorders such as depression, the intense self-reflection involved in self-focused rumination can reflect individual's avoidance of situations or responsibilities, and it often occurs unconsciously or involuntarily[4,6,40]. However, most research on reappraisal and rumination has been conducted in self-focused contexts, and the extent to which results can be applied to interpersonal contexts, especially parenting, is unclear. Longitudinal profiles of parental reappraisal and rumination can tackle questions on their coexistence, address how such coexistence in turn is related to family correlates, and reveal habitual differences in the use of self- and child-focused strategies."

10. *On p.6, authors cite Teuber et al. (2025), who used the same data, as a study that found parents' well-being influenced how they attempt to modulate their children's emotions. Did this study's methods align with the use of this causal language?*

    Authors' response: Thank you for pointing out this example of causal language. As previously mentioned, we carefully checked the entire manuscript for causal language and revised the phrasing where needed. We removed this particular sentence while shortening the manuscript, as we now include a new section earlier in the manuscript that explicitly addresses the evidence on how parents regulate themselves and engage in emotion-related parenting practices.

11. *Can authors expand on p.6 about how they think parental self- or child-focused rumination could be helpful in everyday parenting life?*

    Authors' response: Thank you for this question. We have added remarks on how reappraisal and rumination can be helpful or unhelpful and also restructured the paragraph on reappraisal and rumination and hope it is now easier to understand.

    Changes in manuscript: "In everyday parenting life, however, reappraisal and rumination can sometimes deviate from their habitual associations and may be helpful in some situations and

unhelpful in others[12]. Despite the consistent benefits of self-focused reappraisal, it can, for example, be less effective in situations of high-emotionality or when cognitive resources are limited[37–39]. Self-focused rumination, on the other hand, has been described by individuals as a strategy to process and reflect on negative emotions to feel more in control of a situation[4,40]. Nevertheless, given its close link to disorders such as depression, the intense self-reflection involved in self-focused rumination can reflect individual's avoidance of situations or responsibilities, and it often occurs unconsciously or involuntarily[4,6,40]. However, most research on reappraisal and rumination has been conducted in self-focused contexts, and the extent to which results can be applied to interpersonal contexts, especially parenting, is unclear. Longitudinal profiles of parental reappraisal and rumination can tackle questions on their coexistence, address how such coexistence in turn is related to family correlates, and reveal habitual differences in the use of self- and child-focused strategies."

12. *The latter half of the paragraph spanning page 8-9 is a bit confusing—can authors explain what they mean that "child-focused emotional competencies, including child-focused ER, have been identified as a vulnerability factors for some parent populations and not associated for others", and that "Regulating their children can be very effortful and exhausting for parents with their self-focused ER potentially being central for their well-being, although this effect might not be the same for children's well-being."*

    Authors' response: Thank you very much for this remark. We have considerably shortened the section on correlates of parent and child adjustment to address previous review comments and to keep the article more focused on the key emotional concepts. We therefore decided to delete this part of the manuscript, as it did not seem central to our argument and, as you noted, was not clearly phrased. We included the shortened paragraph for you below.

    Changes in manuscript: "Patterns of parents' self- and child-focused emotion regulation are likely interconnected with family well-being and relationship closeness, with prior work predominantly focusing on links to child development[8,52,64]. Parents' self-focused emotion regulation and parenting practices are robustly tied to how children regulate their own emotions, which in turn is closely linked to their internalizing problems, characterized by inwardly oriented affective disruptions[7,41,65,66]. Overall, parental self- and child-focused emotion regulation has been repeatedly associated with child adjustment[8], which presumably encompasses academic achievement during adolescence[32,47,67]. Less attention has been given to parental correlates including parental exhaustion (i.e. persistent parenting-related stress and an early stage of parental burnout[68,69]), but evidence links parental exhaustion to lower self-focused reappraisal (for a meta-analysis, see [56]), higher self-focused rumination[71], and disruptions in child development[23,72,73]. Lastly, parent–child relationship closeness may be associated with parental self-and child-focused emotion regulation, as higher emotion regulation skills are related to more secure attachment[74,75], and authoritative parenting characterized by warmth and

structure[28,76], whereas self-focused emotional instability has been linked with parental distancing and fewer or lower quality parent–child interactions[21,77]."

13. *For authors point on p.9 that "More concretely, parents' stress responses and self-focused ER are robustly tied to how children regulate their own emotions, which in turn is closely linked to their internalizing problems" I recommend adding citations. One helpful review might be Bariola et al. (2011).*

    Bariola, E., Gullone, E., & Hughes, E. K. (2011). Child and adolescent emotion regulation: The role of parental emotion regulation and expression. Clinical Child and Family Psychology Review, 14(2), 198–212. https://doi.org/10.1007/s10567-011-0092-5

    Authors' response: Thank you for the very helpful citation. When shortening the Introduction, we combined this sentence with the previous one to make it more to the point. We also included the citation of Bariola et al. (2011), which is indeed very relevant here.

    Changes in manuscript: "Patterns of parents' self- and child-focused emotion regulation are likely interconnected with family well-being and relationship closeness, with prior work predominantly focusing on links to child development[8,52,64]. Parents' self-focused emotion regulation and parenting practices are robustly tied to how children regulate their own emotions, which in turn is closely linked to their internalizing problems, characterized by inwardly oriented affective disruptions[7,41,65,66]."

14. *As mentioned above, the systematic review Edler & Valentino (2024) could also be helpful for supporting the point on p.9 that parental self-focused ER is linked to emotion-related parenting behaviors specifically.*

    Authors' response: Thank you for pointing out the reference. We incorporated it into the sentence as you suggested. However, in line with other review comments, this section has now been moved to the beginning of the manuscript and expanded to include additional meta-analyses and reviews on parental self-focused emotion regulation and emotion-related parenting behaviors. We included the revised section below.

    Changes in manuscript: "Consider, for instance, an adolescent who comes home upset after receiving a poor report card and is facing the possibility of repeating the year. The parent may attempt to regulate their own negative emotions (e.g., disappointment or frustration) while also helping the child downregulate their emotional distress. These mechanisms are classified under the concept of emotion regulation, which refers to the process by which individuals modify emotional states in pursuit of specific goals[10,11]. Such regulatory goals can be primarily directed toward one's own emotions (self-focused emotion regulation) or another person's emotions (other-focused emotion regulation)[12], with these goal directions being activated separately or coactivated. The literature on parental self- and child-focused emotion regulation is closely

intertwined with overlapping concepts such as coregulation, emotion socialization and interpersonal emotion regulation[13], where prior research indicates interrelations between how parents regulate their own and their children's emotions[14,15]. More precisely, parents with higher self-focused emotion regulation skills or capacities are more likely to respond supportively to children's emotions, be more involved and sensitive in their parenting, and use fewer negative and unsupportive practices, including hostility and harshness[8,16–18]. Conversely, parents who engage more in self-focused emotion dysregulation tend to use more non-supportive and disengaged regulation practices and are more likely to engage in child maltreatment[8,17,19–22]."

15. *Is there any empirical evidence that authors could cite for their point on p.10 that "Interestingly, closer parent–child relationships often involve more frequent interactions, which also include child-focused rumination"?*

    Authors' response: Thank you for pointing that out! We are not aware of any empirical evidence, as this was based on our theoretical considerations. We have therefore removed the sentence from the Introduction and incorporated a clearer, explicitly speculative version in the Discussion, where we engage with similar ideas.

    Changes in manuscript: "One possibility is that child-focused reappraisal compensates for the potential negative effects of child-focused rumination. Furthermore, a closer parent–child relationship might involve more frequent interactions, which could also include child-focused rumination. Accordingly, the use of child-focused emotion regulation might demonstrate parental attempts to assist their child with negative emotions, which in itself demonstrates engagement and care. Maintaining contact and communication with the child may be the critical factor for the parent–child relationship, even if regulation strategies are not always optimally chosen."

16. *On p.11, it would be helpful for authors to cite the prior work they refer to on ER profiles in parenting. Same with authors' statement that there is moderate stability in self-focused ER and parenting behaviors.*

    Authors' response: Thank you for your comment. In accordance with the Journal's guidelines and to improve concision, we combined the two paragraphs describing the current study into one and removed theoretical elaborations already covered in the Introduction. Below, we provided the revised paragraph.

    Changes in manuscript: "The aim of the current study was to identify subgroups of parents' self- and child-focused emotion regulation (RQ1) and investigate their stability (RQ2). We expected profiles characterized by high self- and child-focused reappraisal combined with low self- and child-focused rumination and, conversely, low reappraisal combined with high rumination.

Furthermore, we expected parents to show relatively stable patterns in their habitual use of self- and child-focused emotion regulation over time. In RQ3, we investigated associations between emotion regulation profiles and parent- and child-focused correlates. We hypothesized that the identified profiles would vary by parents' educational level and gender. However, given the exploratory nature of our analyses and the mixed findings in the literature regarding group differences in reappraisal and rumination[3,48,59], we did not specify the direction of these differences. Furthermore, we hypothesized that profile membership predicts parental exhaustion, children's internalizing problems, children's academic achievement, and the parent–child relationship. Profiles characterized by higher levels of self- and child-focused reappraisal were expected to be associated with lower parental exhaustion and children's internalizing problems but higher children's academic achievement and parent–child relationship. By contrast, profiles characterized by greater use of self- and child-focused rumination were expected to be associated with higher parental exhaustion and children's internalizing problems and lower children's academic achievement and parent–child relationship closeness."

17. *Did authors have hypotheses about the direction of association between profiles and parental education/gender?*

Authors' response: Thank you for the question. Due to the inherently exploratory nature of the analyses, we did not hypothesize specific directions for the SES and gender-related hypotheses. We have therefore clarified this in the manuscript.

Changes in manuscript: "We hypothesized that the identified profiles would vary by parents' educational level and gender. However, given the exploratory nature of our analyses and the mixed findings in the literature regarding group differences in reappraisal and rumination[3,48,59], we did not specify the direction of these differences."

18. *Could authors report more detail about the sample demographics, if possible? 62.91% of parents had a university level education, what about the rest of the sample? Was information gathered on family income and family structure (e.g., single parent status)?*

Authors' response: We appreciate the comment and have added additional sample demographics recorded in the survey, including additional information on households' SES and family and partnership characteristics.

Changes in manuscript: "The sample was gender-balanced with 539 participants (52%) identifying as female. Participants' identified race was for 69.12% white (*n* = 723), 17.59% African American/Black (*n* = 184), 4.40% Asian (*n* = 46), 4.88% Mixed (*n* = 51), 2.29% Other (*n* = 24), and for 1.72% race was not reported (*n* = 18). Sixteen participants (1.53%) reported that their highest educational level was below secondary school, 215 (20.55%) reported secondary school, 130 (12.43%) reported an associate's degree, 449 (42.93%) reported a bachelor's

degree, 184 (17.59%) reported a master's degree, 25 (2.39%) reported a PhD, and 27 (2.58%) reported another level of education. Additionally, we coded households' Highest International Socio-Economic Index of Occupational Status (HISEI)[78,79], which had a mean of 64.65 (*SD* = 16.95, min = 15.35, max = 88.70). We recorded parents' relationship status, with 871 parents responding to be married or in a committed relationship (83.27%), and 175 responding not to be (16.73%). Of these parents, 844 reported living in the same household as their partner (80.69%), 27 reported they did not (2.58%), and 175 provided no response (16.73%). Throughout the questionnaire, participants were instructed to refer to their youngest child in Grades 6–9. According to parent report, 476 of these children identified as female (45.47%), 570 identified as male (54.42%), and none identified as gender diverse. In the sample, 1,010 parents reported living in the same household as their child (96.56%), whereas 36 reported they did not (3.44%). A total of 29.83% of the children were in Grade 6 (n = 312), 18.54% in Grade 7 (n = 194), 16.92% in Grade 8 (n = 177), and 21.13% in Grade 9 (n = 221). A total of n = 237 participants (22.66%) indicated that their child had special needs under *the Individuals with Disabilities Education Act*[80] categories."

19. *What was the attrition from T1 to T2 and T3?*

Authors' response: Thank you for this question. We did not include a clear outline of attrition and the composition of the datasets, which we have now revised in the manuscript. Because only two outcomes from Time point 3 were used in the present study, the primary dataset consisted of data from Time point 1 and Time point 2. We have added a more detailed overview of the procedure, including dropouts and missing data for the different datasets used in the analyses.

Changes in manuscript: "We collected longitudinal data at three time points (T_1 in September 2023, T_2 in January 2024, T_3 in May 2024), following parents across one school year. After cleaning the data and excluding nonmatchable records, 1,359 participants completed the survey at T_1, and 1,046 participants provided data at T_1 and T_2, indicating an attrition rate of 23.03% (n = 313). Thus, the final sample for the main analyses (T_1–T_2 dataset) consisted of N = 1,046 parents of sixth–ninth graders residing in the US. At the third time point, 875 participants could be matched to the T_1–T_2 dataset. Missing data in the T_1–T_2 dataset were 0.60%, and missing data in the dataset including all three time points were 4.62%."

20. *Why did authors only use 4/6 items from the ERQ cognitive reappraisal subscale?*

Authors' response: Thank you for this question. Because the present study focused on the goal of downregulation negative emotions, we included only the ERQ reappraisal items that explicitly refer to reappraising negative emotions and excluded the two items that refer to positive emotions. We also made this rationale more explicit in the manuscript.

Changes in manuscript: "We included four of the six items from the reappraisal subscale, specifically those aligned with the present study's emotion goal of downregulating negative emotions, for example, "*I control my emotions by changing the way I think about the situation I'm in*" with a 5-point Likert response format ranging from 1 (*strongly disagree*) to 5 (*strongly agree*)."

21. *What was the skew for the academic achievement variable, and for the other variables?*

Authors' response: Thank you for the question! We included the skew for all outcome variables in the respective scale description next to Cronbach's alpha.

Changes in manuscript:
"Parental Exhaustion: (…) and the scale was positively skewed at both time points ($skewness_{T1}$ = 1.44, $skewness_{T2}$ = 1.30).

Children's Academic Achievement: (…) The scale was negatively skewed at both time points ($skewness_{T1}$ = −1.30, $skewness_{T2}$ = −1.30).

Children's Internalizing Problems: (…) and the scale was positively skewed with $skewness_{T3}$ = 0.98.

Parent–child relationship: (…) The scale was negatively skewed with $skewness_{T3}$ = −1.24."

22. *Can authors provide a citation for their point on p.15 that internalizing problems are a proximal outcome of children's self-focused ER? Perhaps Compas et al. (2017), referenced above.*

Authors' response: Thank you for your remark, we included Compas et al. (2017) as a reference here.

23. *Why did authors run a two-factor CFA for child internalizing symptoms?*

Authors' response: Thank you for this question. We conducted a two-factor CFA for internalizing problems because, in the Strengths and Difficulties Questionnaire (SDQ), the internalizing score is derived from two subscales: Emotional Symptoms and Peer Relationship Problems. This was not described clearly enough in the manuscript, so we revised the relevant section to make the procedure more understandable.

Changes in manuscript: "Children's internalizing problems were assessed using the 10-item internalizing problems score from the Strengths and Difficulties Questionnaire (SDQ[84]). Specifically, the SDQ internalizing problems score is the sum of two subscales, Emotional Symptoms and Peer Relationship Problems, each consisting of five items. Selection of this score

was theoretically driven, as internalizing problems are a proximal outcome of children's self-focused emotion regulation[63] and therefore important to investigate in the context of parenting emotion regulation profiles. The score assessed the frequency of emotional symptoms (e.g., *restless, overactive, cannot stay still for long*) and the frequency of peer relationship problems (e.g., *often lies or cheats*). The response format ranged from 0 (*not true*) to 2 (*certainly true*). Cronbach's α indicated acceptable results with α = .80 (T_3), and the scale was positively skewed with $skewness_{T3}$ = 1.01."

24. *Were any residuals between different items allowed to correlate in any of the authors' CFAs or the longitudinal measurement invariance models?*

Authors' response: Thank you for this question. In our CFA and LMI models, we did not allow any residuals to correlate, and residual covariances were fixed to zero by default. We have added a brief statement about it to the manuscript.

Changes in manuscript: "For all CFA and LMI models, covariances were fixed to zero by default."

25. *Authors write on p.16 that the configural, metric, and scalar longitudinal measurement invariance models were compared pairwise. Did authors compare configural to metric, and metric to scalar, using likelihood ratio tests?*

Authors' response: Thank you for this question. Indeed, our description was not very clear and may have been misleading. We used robust maximum likelihood estimation for each longitudinal measurement invariance model (configural, metric, and scalar), and then compared the three models using model fit indices to identify the best-fitting model. To clarify this procedure, we revised the respective section of the manuscript.

Changes in manuscript: "Afterwards, Longitudinal Measurement Invariance (LMI) models were estimated for parental self- and child-focused emotion regulation strategies using robust maximum likelihood estimation. For all CFA and LMI models, covariances were fixed to zero by default. Factor loadings, measurement intercepts, and measurement residuals were constrained stepwise, and configural, metric, and scalar LMI models were compared using model fit criteria, including Akaike's Information Criterion (AIC), Consistent AIC (CAIC), Bayesian Information Criterion (BIC), and adjusted Bayesian Information Criterion (ABIC), such that lower values reflected better model fit[95]. After the model with the best fit was identified, factor score standardizations (*M* = 0, *SD* = 1) were saved in M*plus* for subsequent analyses."

26. *On p.16, authors write "Higher entropy values (0-1)…" and I understand that they mean the range of entropy can be from 0-1, but readers may not know this and think that a 0 or a 1 (vs. a hypothetical negative value) indicates poor class separation.*

Authors' response: Thank you for pointing out this potentially misleading phrasing. We revised the sentence according to your recommendation.
Changes in manuscript: "Entropy values range from 0 to 1, with higher values suggesting greater classification accuracy. A significant LMR-LRT indicates a significant improvement in the current model over a model with one fewer profile."

27. *Authors mention using auxiliary three-step analyses. Is this similar to the Bolck-Croon-Hagenaars method? Or more similar to Vermunt's three-step approach? If authors could elaborate on this analytic choice it would be helpful.*

Authors' response: Thank you for this question. The three-step analyses we conducted in M*plus* are based on Vermunt's three-step approach, which is recommended for incorporating predictors and outcomes in latent transition analysis by Asparouhov & Muthén (2014). We have added the relevant citation of Vermunt and briefly justified the analytic choice in the manuscript.

Changes in manuscript: "RQ3 was investigated by entering the antecedents and outcomes stepwise into the LTA in a stepwise fashion using auxiliary three-step analyses[98,99], as this M*Plus* recommended approach allows predictors and outcomes to be incorporated into the LTA model while correcting for classification error."

28. *It is very interesting that the "reappraisers" were also elevated in child-focused rumination. I wonder if authors could relabel this profile to capture that important nuance?*

Similarly, looking at Figure 2, the self-ruminators were actually somewhat low in self-rumination. Could another important dimension of this grouping be parents' disengagement with self-focused reappraisal and child-focused ER?

Authors' response: Thank you for these comments. We agree that the profiles are more complex, particularly given the discrepancy between self- and child-focused rumination across profiles. According with your mentioned points, we also discussed the profile labels at length to identify alternatives that capture the full complexity of the patterns. However, we were unable to identify other labels that were both sufficiently precise and concise. Although the "Self-ruminators" profile does not show extremely high self-rumination in absolute terms compared with some other elevations (e.g., in reappraisal), it represents the highest level of self-rumination in our sample, which is also reflected in the crossing of the profile lines in the plots. Similarly, in the "Reappraiser" profile, both reappraisal indicators are clearly higher than child-focused rumination, even though this profile also shows the relatively highest level of child-focused rumination across the sample. After further consideration, we could not find profile names that were both more accurate and still concise, so we retained the current labels. Your suggestion to include disengagement was very valuable, but we ultimately refrained from doing so because we assessed only four strategies and were therefore hesitant to generalize our

findings to overall emotion regulation strategy use. We also included this important point in our discussion. However, we would be very happy to incorporate alternative suggestions if you have ideas for more fitting profile names.

Changes in manuscript: “As the name suggests, *self-ruminators* had the highest levels of self-focused rumination compared with the other profiles, but they also reported very low levels of the three other strategies, particularly self- and child-focused reappraisal. This profile may include parents who are highly exhausted and engage in frequent self-focused rumination, leaving them with limited capacities to regulate their emotions using potentially helpful strategies (e.g., self-focused reappraisal). Associated with this exhaustion, parents in this profile may be absorbed by their own emotions, which could reduce their ability to focus on their children’s needs[104] and could explain the low levels of child-focused emotion regulation in this profile, potentially indicating some disengagement from parenting practices. Alternatively, these parents might recognize the maladaptive nature of self-focused rumination and actively try not to transfer it to their children by avoiding child-focused rumination.”

Figure 1

A. Profiles of Parental Self- and Child-Focused Emotion Regulation Strategies at T_1

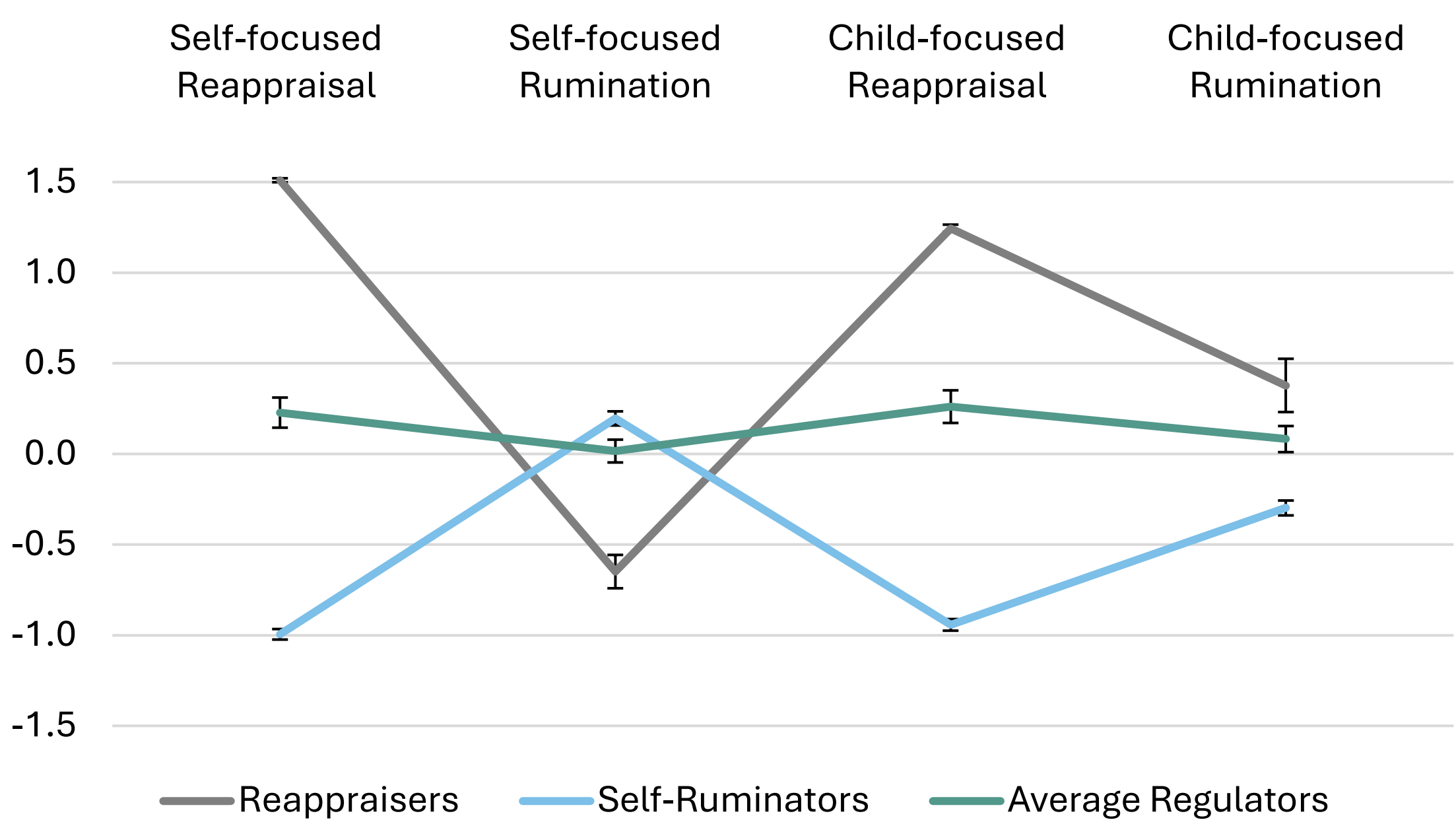

B. Profiles of Parental Self- and Child-Focused Emotion Regulation Strategies at T_2

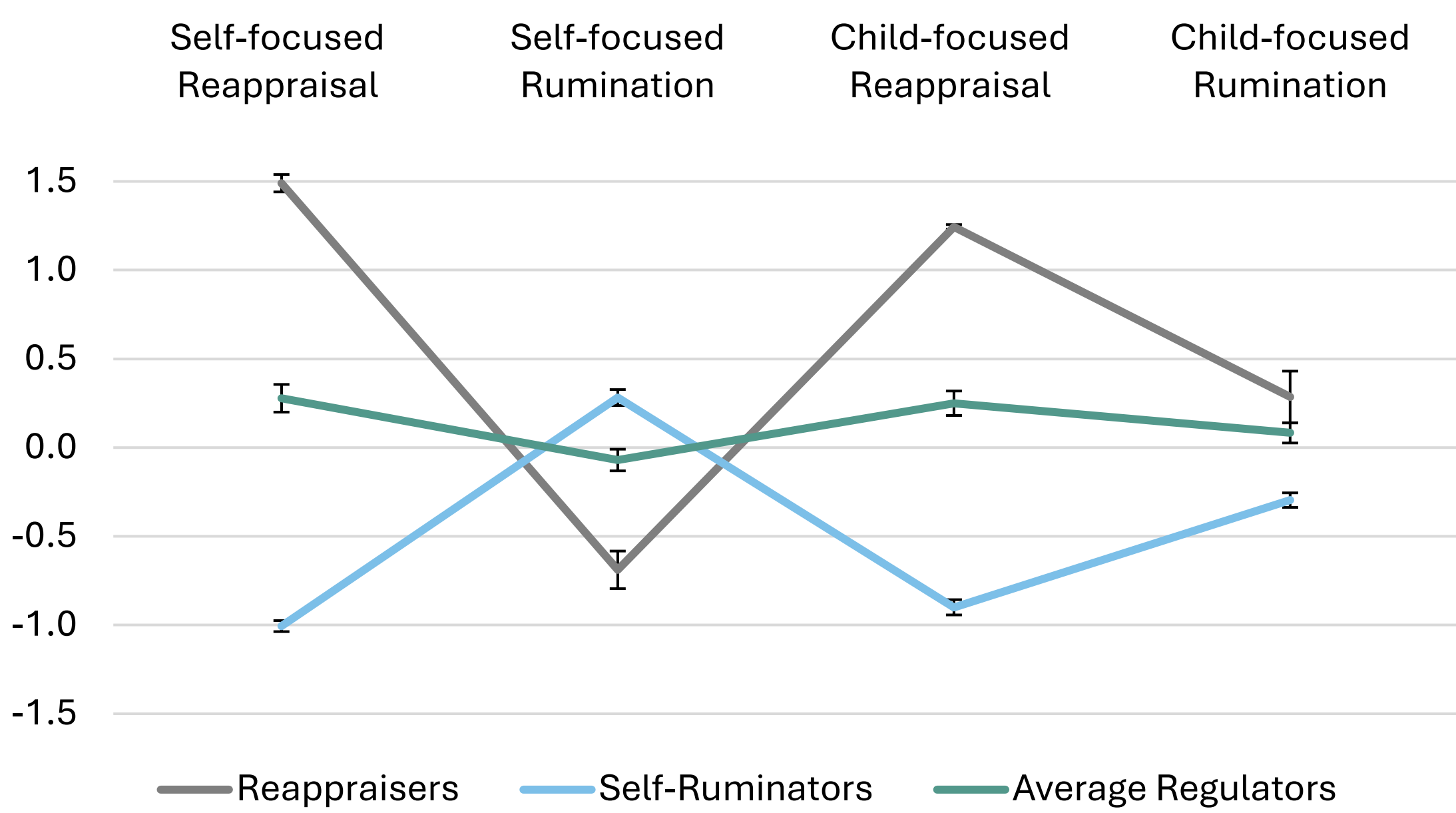

Note. T_1 = time point one; T_2 = time point two; each emotion regulation strategy in the profiles is reported with error bars representing ±1 standard error of the mean, N = 1,046 participants.

29. *Did authors conduct formal pairwise comparisons to conclude that the average regulators had lower self- and child-focused reappraisal compared with the those in the reappraisers profile (p.19), or were these just visual comparisons?*

Authors' response: Thank you for this comment. Previously, our comparisons were primarily based on visual inspection of plots, including error bars. We have now conducted ANOVAs and chi-square tests to compare class demographics, and these analyses indicated the same pattern of results as described in the sentence you noted. We therefore did not rephrase that paragraph, but we added a sentence about the statistical tests and included a new table for the statistical tests (Table 2).

Changes in manuscript: "In Table 2, we report profile demographics for the emotion regulation strategies included in the profiles and the profile correlates. Differences between profiles were tested using analyses of variance (ANOVAs) for continuous variables and chi-square tests for categorical variables."

30. *It's interesting that self-ruminators had the highest stability. At the same time, it looks like a decent number of parents transitioned into the self-rumination profile. In a sense, could this indicate there was some instability in level of self-rumination?*

Authors' response: Thank you for pointing out this change in the profiles. After further discussion, we considered whether it might reflect some instability in the other profiles and/or an overall increase in self-focused rumination across time. When comparing Wave 1 and Wave 2, the mean level of self-focused rumination within the self-ruminator profile increased from 0.20 at Wave 1 to 0.28 at Wave 2. In contrast, the overall mean of self-focused rumination in the full sample was very stable across waves, showing a slight decrease from Wave 1 to Wave 2 (M = 2.46 vs. M = 2.44). We have incorporated this observation into the Discussion.

Changes in manuscript: "The *self-ruminators* profile represented the second largest group and was the only one to increase in size over time (n_{T1} = 264; n_{T2} = 326). These results may reflect rising stress and regulatory demands as the school year progresses, given that T_1 occurred at the start of children's school year. Interestingly, the overall sample mean of self-focused rumination was very stable across both time points, whereas the *self-ruminators* showed a slight increase in self-focused rumination, demonstrating a central advantage of person-centered approaches in identifying subgroup-specific changes that are not captured my aggregate means."

31. *Authors say on p.21 that "Contrary to our hypotheses, child-focused rumination aligned with child-focused reappraisal but at lower levels"—I thought the reappraisers profile was characterized by both relatively higher levels of reappraisal and child-focused rumination?*

Authors' response: Thank you for pointing out this misleading phrasing. You are correct that the reappraisers profile is characterized by relatively higher levels of reappraisal and child-focused rumination, which we attempted to communicate here but expressed suboptimally. We have therefore clarified the wording and hope it is now easier to understand.

Changes in manuscript: "Contrary to our hypotheses, child-focused rumination followed a similar pattern as child-focused reappraisal across profiles, consistent with prior variable-focused analyses showing positive associations between child-focused reappraisal and rumination[45]."

32. *It would be helpful for authors to expand on their point that "These differences are also reflected by the respective measurement scales where self-focused rumination typically includes reflection and brooding subscales, whereas child-focused rumination is assessed through reflection only." Readers less familiar with the rumination literature could benefit from more discussion about the distinction between reflection and brooding.*

Authors' response: Thank you for your comment. We have added further elaboration on these differences in the respective paragraph (outlined below). In response to previous comments, we also expanded the theoretical section on mechanisms of rumination, including reflection and brooding.

Changes in manuscript: "These differences are also reflected in the respective measurement scales, where self-focused rumination typically includes reflection and brooding subscales, which capture negative self-focus and self-blame as the two key mechanisms underlying rumination[4,5]. Child-focused rumination, on the other hand, is assessed through reflection only and potentially weaker because the focus shifts away from the heightened self-focus[4,83]. Reappraisal and rumination might also be more closely related in interpersonal contexts, as the reflective aspect of child-focused rumination could represent parents' attempts to encourage their children to think through their experiences as well as increased involvement. Future research should examine the robustness of our results to better understand similarities and differences in the composition of reappraisal and rumination in self- and child-focused contexts."

33. *I wonder if it would be helpful for authors to list all five of the child-focused rumination items to better interpret the findings.*

Authors' response: Thank you for your comment. Indeed, because child-focused rumination has been less investigated and its conceptualization can differ from self-focused rumination, it is a very good idea to include all items of the scale. For consistency, we also added all items of the child-focused reappraisal scale to the manuscript.

Changes in manuscript: "The items of child-focused reappraisal included "*I help [adolescent's nickname] change their feelings by thinking differently about their current situation*", "*I help [adolescent's nickname] think of a situation in a positive light*", "*I help [adolescent's nickname] see the situation from a different perspective*", "*I help [adolescent's nickname] try to see the positive aspects of a situation that is making them have negative feelings*", and "*I encourage [adolescent's nickname] to think of the positive side to their negative feelings*". The items of child-focused rumination, on the other hand, included "*I help [adolescent's nickname] replay the experience of negative feelings again and again in their mind*", "*I help [adolescent's nickname] replay whatever is making them have negative feelings*", "*I help [adolescent's nickname] think again and again about whatever is making them have negative feelings*", "*I encourage [adolescent's nickname] to think over and over again about why they are having negative feelings*", and "*I help [adolescent's nickname] think about situations that are upsetting or that cause negative feelings over and over again*"."

34. *P.24 of the discussion is another spot where authors use causal language and strongly imply that the profiles are leading to the "outcomes." I think it would be worthwhile for authors to explore the possibility that the outcomes may be contributing to the profiles. E.g., if a child has more internalizing symptoms, or a parent is experiencing more burnout, maybe that contributes to higher self-focused rumination? This is an important direction to consider, especially as it sounds like internalizing symptoms at T1 were examined in association with transitions in profiles from T1 to T2.*

Authors' response: Thank you for your very important comment. We carefully checked the discussion to refrain from using causal language. Additionally, we included your example and evolved it to make understandable how the profiles and correlates might be closely intertwined and most likely bidirectionally influence each other.

Changes in manuscript: "Nevertheless, parents' habitual patterns of self-focused reappraisal and rumination are bidirectionally associated with parents' healthy functioning[24,52,72]. Therefore, it is important to consider that our measured correlates may also contribute to the profiles. For example, higher child internalizing problems or greater parental exhaustion might increase parents' self-focused rumination, whereas in the *reappraisers* profile, lower parental exhaustion and fewer child internalizing problems might be linked to parents' greater capacity to use self- and child-focused reappraisal[38]. Similarly, parents have been posited to experience lower distress when their child becomes better regulated[72]."

35. *On p.24 authors cite Zimmer-Gembeck et al. (2022) and say that "Previous research has also shown that parental self-focused ER is not necessarily directly associated with parenting behavior but is related to children's socioemotional adjustment." This is not what the meta-analysis found; parental ER was associated directly with parenting behavior. See also Crandall et al. (2015), Edler & Valentino (2024), Zitzmann et al. (2024).*

    Authors' response: Thank you very much for correcting us regarding this misleading statement. We have deleted the sentence from the revised manuscript.

36. *On p.25, adding the world parental before "child-focused ER" would be helpful for this sentence: "Also, children's self-focused ER can influence academic achievement, but evidence of child-focused ER remains limited."*

    Authors' response: Thank you for this remark. We agree that it enhances clarity, and we have incorporated your suggestion accordingly.

37. *Do authors expect that findings would have been similar if they had used a non-academic related stem for the parental child-focused ER efforts?*

    Authors' response: This is a very interesting question. Because the items were framed to capture parents' habitual use of child-focused emotion regulation strategies, we do not necessarily expect the results to differ if non-academic stems had been used. However, because we are not aware of studies that have tested this directly, the answer to that question is uncertain. Some research suggests that the effectiveness of emotion regulation strategies can vary depending on whether the context is academic or non-academic, which raises the possibility that parents'

strategy use might also differ across such contexts. In response to this point, we have added a statement to the limitations and highlighted it as a direction for future research.

Changes in manuscript: "For child-focused emotion regulation, we adapted the items to academic achievement contexts, which may have introduced limitations such as a potential misalignment between self- and child-focused emotion regulation that could have influenced profile membership and the profiles' associations with antecedents and outcomes. Some research suggests that strategy effectiveness differs between academic and non-academic contexts[42], which could imply that parents' child-focused emotion regulation strategy use also varies by context. Therefore, future studies should directly compare child-focused strategy use across academic and non-academic situations."

38. *I think there is a typo on p.26, when authors say "Future research should include a broader range of studies, incorporating strategies such as problem-solving and avoidance," do they mean strategies instead of studies?*

    Authors' response: Thank you for the comment. Indeed, this was a typo which we corrected now.

    Changes in manuscript: "Future research should include a broader range of strategies, including problem-solving and avoidance, which have recently been shown to be relevant for achievement regulation."

39. *Hajal & Paley (2020) is a relevant study for the authors' point on p.28 about intervening via parental emotion regulation to support child development.*

    Authors' response: Thank you for pointing out this reference. However, we deleted this sentence from our discussion, as the journal guidelines state that intervention suggestions should not be included unless interventions were explicitly tested.
