# Supplementary material for: Longitudinal profiles of parental self- and child-focused emotion regulation
Source: Commun Psychol. 2026 May 16;4:115. doi: 10.1038/s44271-026-00469-w (PMC13469115; doi:10.1038/s44271-026-00469-w)
Supplement: Supplementary file 2 [file 44271_2026_469_MOESM2_ESM.pdf]

**Supplementary Materials**

**Table S1**

*Confirmatory Factor Analysis (CFA) for Measures*

| Measure | Time point | $\chi^2/df$ | CFI | RMSEA [90% *CI*] | SRMR |
|---|---|---|---|---|---|
| Self- and Child-Focused Emotion Regulation (four-factor CFA) | 1 | 418.14***/146 | .96 | .04 [.04, .05] | .04 |
| | 2 | 359.47***/146 | .97 | .04 [.03, .04] | .04 |
| Parental Exhaustion (one-factor CFA) | 1 | 54.99***/27 | .99 | .03 [.02, .04] | .02 |
| | 2 | 47.98***/27 | .99 | .03 [.02, .04] | .02 |
| Children Internalizing Problems (two-factor CFA) | 3 | 185.86***/34 | .90 | .07 [.06, .08] | .05 |
| Parent–Child Relationship (one-factor CFA) | 3 | 1.07***/2 | 1.00 | .00 [.00, .04] | .01 |

*Note.* Models were estimated with robust maximum-likelihood (MLR); all $\chi^2$ and fit indices reported are Satorra–Bentler scaled; $\chi^2$ = chi-square value; df = degrees of freedom; CFI = comparative fit index; RMSEA = root mean square error of approximation; CI = confidence interval; SRMR = standardized root mean square residual; *$p \leq .05$, **$p \leq .01$, ***$p \leq .001$.

**Table S2**

*Longitudinal Measurement Invariance (LMI) Models of Parental Self- and Child-focused ER Strategies*

| LMI Model | $\chi^2/df$ | CFI | RMSEA [90% *CI*] | SRMR | AIC | CAIC | BIC | ABIC |
|---|---|---|---|---|---|---|---|---|
| Configural LMI | 1144.86***/ 618 | .97 | .03 [.03, .03] | .04 | 92184.08 | 93142.47 | 92981.47 | 92470.11 |
| Metric LMI | 1182.18***/ 637 | .97 | .03 [.03, .03] | .04 | 92187.18 | 93032.47 | 92890.47 | 92439.46 |
| **Scalar LMI** | **1214.55***/ 656** | **.97** | **.03 [.03, .03]** | **.04** | **92180.94** | **92913.13** | **92790.12** | **92399.46** |

*Note.* Selected model in bold font; LMI = longitudinal measurement invariance; $\chi^2$ = chi-square value; df = degrees of freedom; CFI = comparative fit index; RMSEA = root mean square error of approximation; CI = confidence interval; SRMR = standardized root mean square residual; AIC = Akaike information criterion; CAIC = consistent AIC; BIC = Bayesian information criterion; ABIC = sample size adjusted BIC; *$p$ ≤ .05, **$p$ ≤ .01, ***$p$ ≤ .001.

**Table S3**

*Model Fit Indices for Profile Solutions at $T_1$ and $T_2$*

| Time point | Profile number | LL | Scaling | fp | AIC | CAIC | BIC | ABIC | Entropy | LMR |
|---|---|---|---|---|---|---|---|---|---|---|
| 1 | 1 | –5652.65 | 1.05 | 8 | 11321.31 | 11368.93 | 11360.93 | 11335.52 | – | – |
| 1 | 2 | –5346.42 | 1.27 | 17 | 10726.84 | 10828.04 | 10811.04 | 10757.05 | .99 | 602.83*** |
| **1** | **3** | **–5073.46** | **1.15** | **26** | **10198.91** | **10353.68** | **10327.68** | **10245.10** | **.82** | **537.35*** ** |
| 1 | 4 | –4902.99 | 1.22 | 35 | 9875.98 | 10084.33 | 10049.33 | 9938.16 | .83 | 335.56*** |
| 1 | 5 | –4799.88 | 1.19 | 44 | 9687.78 | 9949.70 | 9905.70 | 9765.95 | .85 | 202.96** |
| 1 | 6 | –4730.04 | 1.20 | 53 | 9566.09 | 9881.58 | 9828.58 | 9660.25 | .82 | 137.50** |
| 2 | 1 | –5726.53 | 1.01 | 8 | 11469.05 | 11516.67 | 11508.67 | 11483.26 | – | – |
| 2 | 2 | –5446.71 | 1.25 | 17 | 10927.41 | 11028.61 | 11011.61 | 10957.61 | .89 | 550.84*** |
| **2** | **3** | **–5194.33** | **1.41** | **26** | **10440.65** | **10595.42** | **10569.42** | **10486.84** | **.83** | **496.82** ** |
| 2 | 4 | –5025.54 | 1.18 | 35 | 10121.07 | 10329.42 | 10294.42 | 10183.25 | .84 | 333.24*** |
| 2 | 5 | –4930.20 | 1.31 | 44 | 9948.40 | 10210.32 | 10166.32 | 10026.57 | .82 | 151.25 |
| 2 | 6 | –4865.12 | 1.33 | 53 | 9836.23 | 10151.73 | 10098.73 | 9930.39 | .81 | 139.63 |

*Note.* Selected models in bold font; LL = log-likelihood model; Scaling = scaling correction factor for robust maximum-likelihood estimation; fp = number of free parameters; AIC = Akaike information

criterion; CAIC = consistent AIC; BIC = Bayesian information criterion; ABIC = sample size adjusted BIC; LMR = Lo-Mendell-Rubin adjusted likelihood ratio test; $*p \le .05$, $**p \le .01$, $***p \le .001$.

**Figure S1**

*Elbow Plot of AIC and BIC at $T_1$*

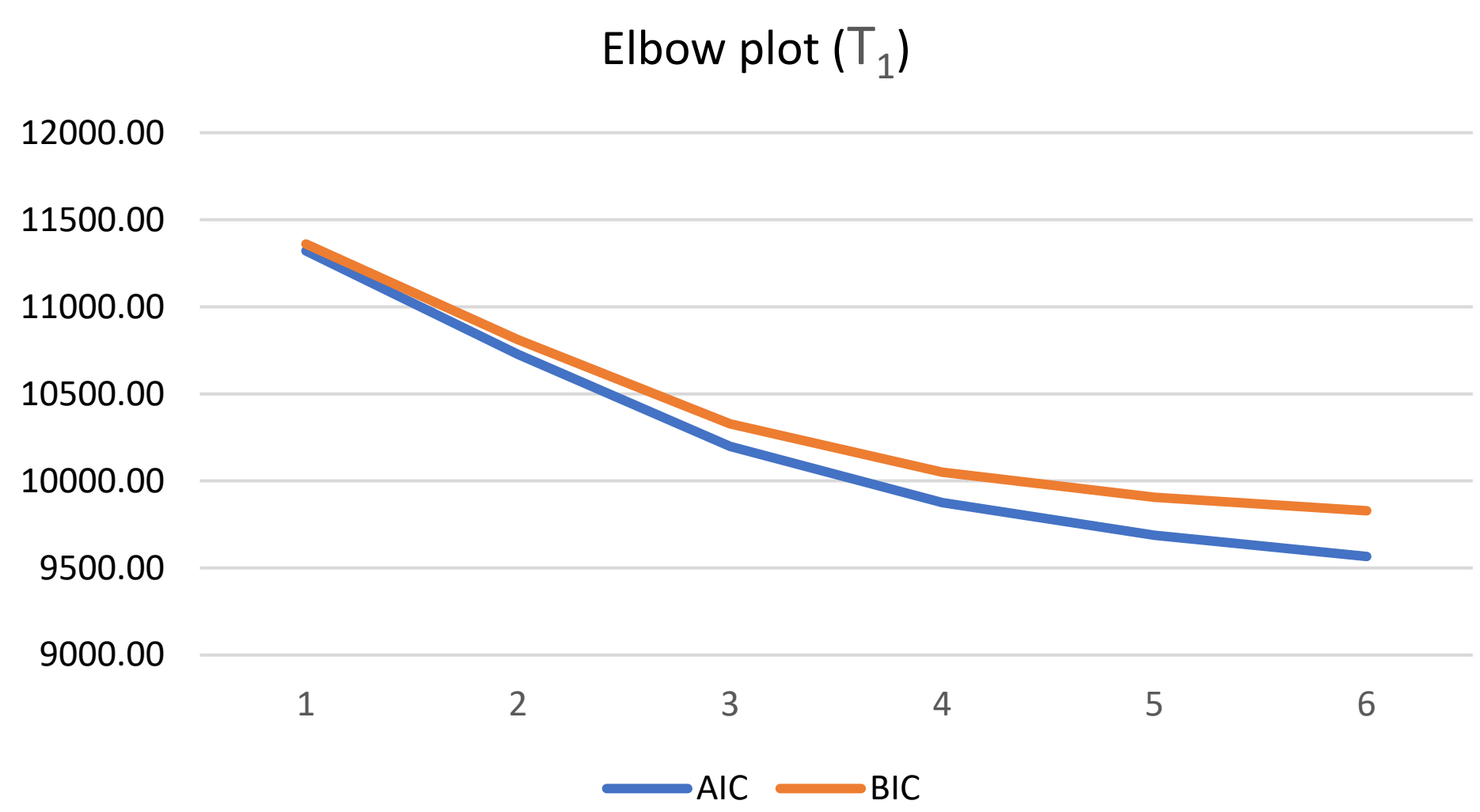


*Note.* AIC = Akaike information criterion; BIC = Bayesian information criterion; $T_1$ = time point one.

**Figure S2**

*Elbow Plot of AIC and BIC at $T_2$*

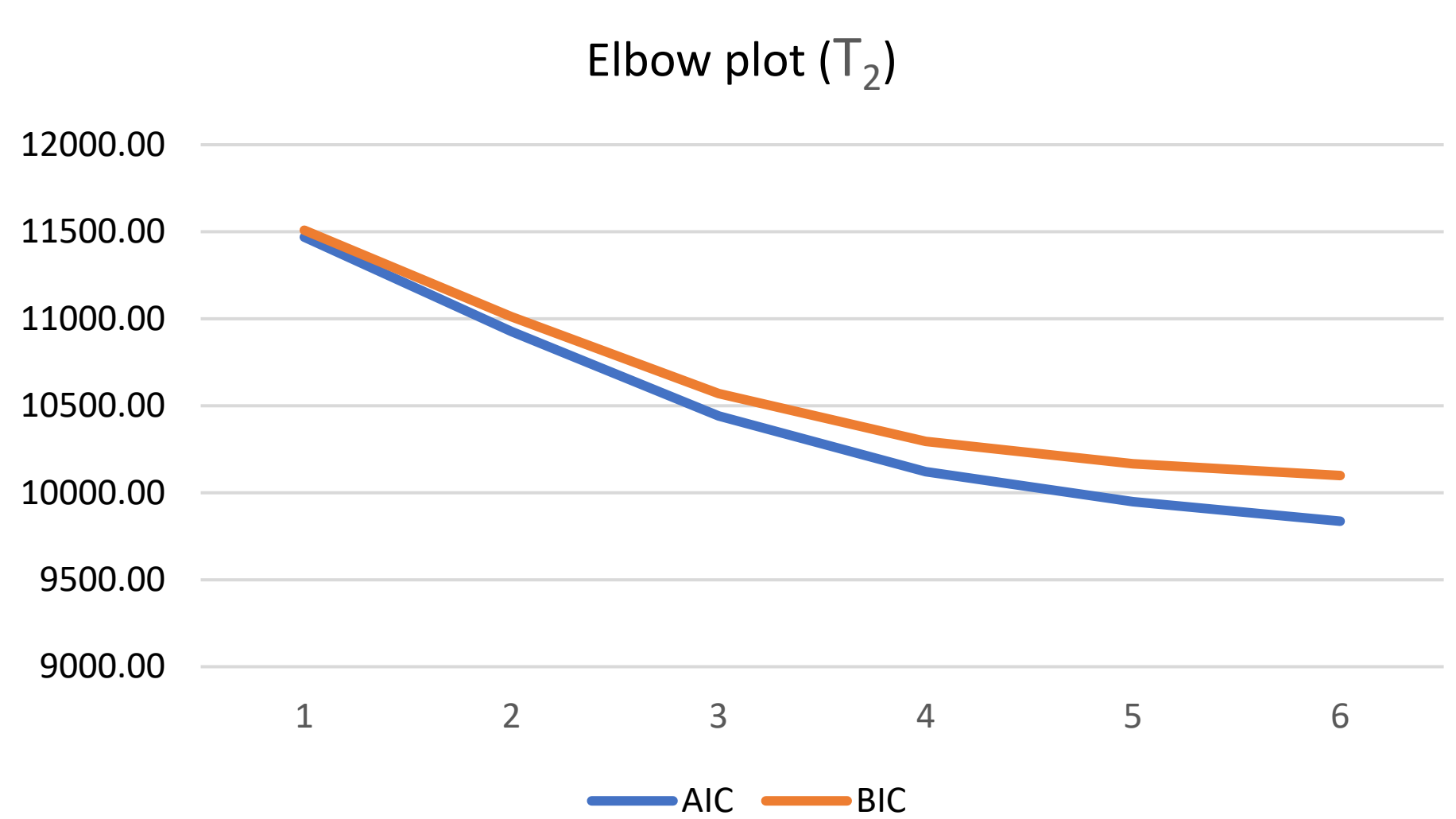


*Note.* AIC = Akaike information criterion; BIC = Bayesian information criterion; $T_2$ = time point two.

**Table S4**

*Longitudinal Latent Profile Analysis (LPA) Models and Latent Transition Analysis (LTA) Models*

| Model | LL | Scaling | fp | AIC | CAIC | BIC | ABIC | Entropy |
|---|---|---|---|---|---|---|---|---|
| ***Longitudinal LPA*** | | | | | | | | |
| Configural Similarity | –10268.28 | 1.23 | 52 | 20640.55 | 20950.09 | 20898.09 | 20732.93 | .81 |
| Structural Similarity | –10282.91 | 1.23 | 40 | 20645.82 | 20883.93 | 20843.93 | 20716.88 | .81 |
| Dispersion Similarity | –10288.69 | 1.39 | 28 | 20633.39 | 20800.07 | 20772.07 | 20683.13 | .81 |
| **Distributional Similarity** | **–10291.49** | **1.46** | **26** | **20634.98** | **20789.75** | **20763.75** | **20681.17** | **.81** |
| **LTA** | **–1481.23** | **0.75** | **8** | **2978.46** | **3027.08** | **3018.08** | **2992.67** | **.85** |
| ***Predictive Similarity Analyses*** | | | | | | | | |
| Gender[1] | –1480.03 | 0.84 | 12 | 2984.07 | 3055.50 | 3043.50 | 3005.38 | .86 |
| **Gender[2]** | **–1480.09** | **0.80** | **10** | **2980.19** | **3039.72** | **3029.72** | **2997.95** | **.86** |
| Educational Level[1] | –1480.55 | 0.87 | 12 | 2985.09 | 3056.53 | 3044.53 | 3006.41 | .86 |
| **Educational Level[2]** | **–1480.60** | **0.80** | **10** | **2981.20** | **3040.73** | **3030.73** | **2998.97** | **.86** |
| ***Final Explanatory Similarity Analyses*** | | | | | | | | |
| Academic Achievement[1] | –5717.64 | 1.03 | 16 | 11467.29 | 11562.53 | 11546.53 | 11495.71 | .86 |
| **Academic Achievement[2]** | **–5719.01** | **1.17** | **13** | **11464.03** | **11541.41** | **11528.41** | **11487.12** | **.86** |
| Parental Exhaustion[1] | –5039.99 | 1.07 | 16 | 10111.97 | 10207.22 | 10191.22 | 10140.40 | .86 |
| **Parental Exhaustion[2]** | **–5040.57** | **1.24** | **13** | **10107.14** | **10184.53** | **10171.53** | **10130.24** | **.86** |
| **Child Internalizing Problems[1]** | **–1859.30** | **0.84** | **12** | **3742.59** | **3814.03** | **3802.03** | **3763.91** | **.86** |
| **Parent–Child Relationship[1]** | **–2258.29** | **0.94** | **12** | **4540.59** | **4612.02** | **4600.02** | **4561.91** | **.86** |

*Note.* Bold font indicates selected and final models; LL = log-likelihood model; Scaling = scaling correction factor for robust maximum-likelihood estimation; fp = number of free parameters; AIC = Akaike information criterion; CAIC = consistent AIC; BIC = Bayesian information criterion; ABIC = sample size adjusted BIC; predictive similarity and final explanatory similarity analyses were computed and compared in two steps when more than one time point was included with [1]freely estimated variances and [2]constrained variances.
